# Supplementary material for: Lineage-specific patterns of chromosome evolution are the rule not the exception in Polyneoptera insects
Source: Proc Biol Sci. 2020 Sep 30;287(1935):20201388. doi: 10.1098/rspb.2020.1388 (PMC7542826; doi:10.1098/rspb.2020.1388)
Supplement: supplementary figures and tables [file rspb20201388supp1.pdf]

Lineage-specific patterns of chromosome evolution are the rule not the exception in Polyneoptera insects

Terrence Sylvester[1], Carl E. Hjelmen[1], Shawn J. Hanrahan[2], Paul A. Lenhart, J. Spencer Johnston[2], and Heath Blackmon[1]

[1] Department of Biology, Texas A&M University, College Station, TX 77843, USA

[2] Department of Entomology, Texas A&M University, College Station, TX 77843, USA

DOI: 10.1098/rspb.2020-1388

Supplementary Materials

Supplementary Methods

Chromosome data

We downloaded all available chromosome data for the clade Polyneoptera from the Tree of Sex database (1, 2). To supplement this data, we performed literature searches for each order in Polyneoptera. Briefly, we combined order names with the terms "cytogenetic", "cytotaxonomic", "karyotype", and "sex chromosome system". For each species in our dataset, we made an attempt to score three traits: chromosome number, sex chromosome system (SCS), and reproductive mode (sexual vs. asexual). In cases where there were multiple records for a species that had different values, we retained all reported values. This process yielded a final dataset of 823 records, 773 of which are unique taxa, with the remaining records representing species that have variability in one of the recorded traits. There are 753 taxa which had data for SCSs, 814 which had data for chromosome number and 749 taxa which had data for both SCS and chromosome number (Table S1). We have also created an interactive database that allows users to explore, plot, and download all Polyneoptera karyotype data which can be accessed at www.karyotype.org.

Phylogenetic data

We used PyPHLAWD to retrieve sequence clusters and used clusters which had at least 100 species for our analysis (3). These included three mitochondrial genes (COI, COX2 and ND4) and three nuclear regions (18S and two regions of the 28S gene). We removed duplicate sequences and retained the longest example for each species using the function FastaFilter in the R package evobiR (4). To maximise the overlap between our trait and sequence datasets we first found all species-level exact matches in both datasets. Next, we looked for genus-level matches that lacked species-level matches. For each of these genus-level matches, we retained the longest sequence for each locus from any species in the genus and used these sequences to act

as a tip representing the genus rather than any single species. This process of maximising the overlap between the two datasets created 57 exemplar taxa (genera tips).

We used the online MAFFT version 7 server (https://mafft.cbrc.jp/alignment/software/) under default settings to align all sequences (5). For the aligned RNA coding sequences, we used GBLOCKS v 0.91b to remove hypervariable regions (6). When running GBLOCKS, we used default settings with the exception of the allowed gap positions argument which was set to maximum. For the 18S sequence cluster, we also set the minimum block length to 6 to retain a greater proportion of the alignment. For the protein-coding genes, we manually adjusted the starting position of the alignments to maintain the reading frame. Using the supermatrix function in the R package evobiR, individual gene alignments were then concatenated into a supermatrix with 7380 sites (4).

The presence of rogue taxa (taxa that have an inconsistent placement in a set of phylogenetic trees) can produce unreliable rate inferences similar to that found in analyses of supertrees (7, 8). To identify the presence of rogue taxa, we generated 100 maximum likelihood rapid bootstrap trees using RAxML v 8.2.10 implemented in CIPRES Science Gateway (9, 10). Using these trees, we calculated the taxonomic instability index as implemented in Mesquite v 3.51 (11). When we examined taxonomic instability indices (TII), we found that a score of 4870 was shared by several species and after that each additional species had increasing TII (Figure S1). We identified 16 taxa whose taxonomic instability index was higher than 4870 and removed them from subsequent analysis. Our final alignment contained 232 taxonomic units with 73% missing data (accession numbers are provided in Table S2).

We used BEAST v 2.5 (12) to infer time-calibrated phylogenies under a relaxed lognormal clock, a birth-death model, and GTR + G as the nucleotide substitution model. The mitochondrial and nuclear coding genes were partitioned into all three coding positions. We used previous estimates for the ages of seven nodes (Table S3) in our phylogeny drawn from a previous study of divergence times across insects (13). For each of these calibration points, we used a normal distribution. The upper and lower bounds of the calibration points (95th and 5th percentiles respectively) were placed according to the confidence intervals as presented in Misof *et al.,* (13). We conducted two independent runs, each for 100 million generations. The convergence of these two independent runs was evaluated using Tracer v 1.7 (14). The initial 50% of each MCMC run was discarded as burnin and 50 phylogenetic trees were randomly sampled from the post-burnin period of each run to construct a posterior distribution of 100 trees used for trait analyses described below.

The presence of parthenogenetic taxa in our dataset allows us to ask how reproductive mode affects chromosome number evolution. However, the parthenogenetic mode of reproduction is only present in Phasmatodea. Therefore, we built a second phylogeny, for Phasmatodea, that included more species from our trait dataset. To do this we supplemented the sequence data from PyPHLAWD with additional data located using the PhyLoTA web server (15). This increased our samples from 28 species, which we had in Polyneoptera dataset, to 41 species. This new dataset included three mitochondrial genes (16s, COI, COX2) and three nuclear genes (18S, 28S and H3). The new alignment consisted of 57% missing data. We inferred time-calibrated phylogenies as described above. However, dating was based on a prior applied to the divergence between the orders Phasmatodea and Embiidina (Table S3).

Genome size estimation

Existing data was supplemented with new genome size estimates for 60 polyneoptera species using the flow cytometric method (Table S4) (16). Briefly, neural tissue was dissected from each insect and placed into Galbraith buffer for co-preparation with the *Periplaneta americana* standard (1C = 3,338 Mbp) (17). Tissue was ground gently with a Kontes 'loose' A pestle approximately 10-15 times before filtered through 41-micron filter. Samples were stained for at least 30 minutes with 25µl of 1mg/ml Propidium iodide before running through a Partec Cyflow SL 3 Flow cytometer with a 532 nm green laser. Samples were run to assure at least 1,000 nuclei under each 2C peak.

Choice of software for modeling chromosome number evolution

There has been a proliferation of probabilistic models of chromosome number evolution in the last several years. Each of these has slightly different goals and approaches. The first of these was chromEvol which focuses on estimates of rates and ancestral conditions within single clades and allows for Bayesian and maximum likelihood approaches to parameter estimation (18, 19). The base model implemented in chromEvol has been expanded to more complex models like chromeploid. Chromeploid focuses on evolution of polyploidy and interactions between chromosome number and binary traits and performs parameter estimation in a maximum likelihood framework (20). Two other extended models have been implemented in a Bayesian framework; ChromeSSE which compares cladogenetic and anagenetic models of evolution (21), and chromePlus which focuses on the interactions between chromosome number and binary traits and allows for the binary trait to impact rates of diversification (22). For the purposes of our investigation chromePlus was the most appropriate because of the flexibility offered within a Bayesian analysis and its ability to account for interactions between chromosome number evolution and binary traits (22). We did not use State-dependent Speciation and Extinction models (SSE) which is implemented in ChromoSSE due to the fact that

non-SSE models perform almost identically to a cladogenetic/anagenetic SSE model (like ChromoSSE) with regard to ancestral state reconstructions (21). Furthermore, we find that rate estimates in SSE and non-SSE models are largely the same (22).

Supplementary results

Chromosome number variation

We find a significant difference in variance in chromosome numbers among the orders of Polyneoptera (Levene's test p-value: 2.2e-16). Blattodea (including Isoptera) (n = 172) had the highest variance in chromosome number (39.32) and Embiidina (n = 8) had the lowest variance in chromosome number (0.41). Orthoptera, despite having 319 records, had a low variance in chromosome number (2.87). We performed Tukey's HSD posthoc test to determine which orders had significantly different variances (tests were considered significant at an alpha of 0.05). We found that Blattodea (including Isoptera) had a higher variance in chromosome number than Embiidina, Mantodea, Orthoptera, Phasmatodea, and Plecoptera. Likewise, Dermaptera had a higher variance in chromosome number than Mantodea and Orthoptera. Finally, Orthoptera had a lower variance in chromosome number than Phasmatodea. Much of these differences in variance are obvious even when looking at the reduced phylogenetic dataset in Fig. 1.

Chromosome Number Ancestral state reconstruction

Prior to the application of probabilistic models of chromosome number evolution the ancestral chromosome number was often assumed to be the most common chromosome number in extant species (23). The approach that we use estimates the probability of possible ancestral chromosome numbers given the observed data, the phylogeny, and rates of fusions, fissions, and polyploidy. We report the estimated ancestral chromosome number in each order, as the most probable value (averaging across all 100 trees from the posterior distribution).
Our analysis of Orthoptera (modal chromosome number 12) suggests six chromosomes is the most probable ancestral state for the order, with a 75.1% probability. However, a chromosome number of three is given a non-trivial probability of 24.5%, which may suggest an early whole-genome duplication in this clade. Support for the lower ancestral state of 3 was concentrated in 19 trees; however, in all other trees, 6 was given the highest probability (Figure S4). This suggests that phylogenetic uncertainty in this clade can have a strong impact on the inference of chromosome number. In Blattodea including Isoptera (modal chromosome number 21), the most probable chromosome number was 13 with a probability of 20.9% followed by 14 and 12 with probabilities 19.5% and 16.4%, respectively. In Blattodea excluding Isoptera (modal chromosome number 19), the most probable chromosome number was 7 with a probability of 11.9% followed by 8 and 9 with probabilities 11.6% and 11.2%, respectively. In Isoptera (modal

chromosome number 21), the most probable chromosome number was 20 with a probability of 35.6% followed by 21 and 22 with probabilities 30.7% and 14.0%, respectively. In Mantodea (modal chromosome number 14), the most probable chromosome number was 8 with a probability of 41.2% followed by 9 and 7 with probabilities 19.2% and 16.1%, respectively. Finally, in Phasmatodea (modal chromosome number 18), the most probable chromosome number was 9 with a probability of 11.7% followed by 10 and 11 with probabilities 11.3% and 10.7%, respectively. All chromosome numbers reported here are haploid counts.

Supplementary discussion

Eusociality and chromosome number

In our analysis of Blattodea (including Isoptera), our results show a significantly lower rate of chromosome number evolution in the subclade Isoptera than the rest of Blattodea (excluding Isoptera). In the past, it has been hypothesised that large rate differences, for instance, those seen among some mammal clades might be explained by differences in effective population size (24, 25). Future work that examines effective population sizes of solitary Blattodea (excluding Isoptera) and eusocial Isoptera might reveal whether the observed rate differences in these lineages is consistent with this earlier hypothesis that large effective population size is associated with lower rates of chromosome number evolution. It should be noted that more recent phylogenetically informed analysis of over 1000 species of mammals showed that female meiotic drive may be a primary driver of chromosome number evolution in mammals (22). Furthermore, the rate difference that we infer among the eusocial and solitary lineages of Blattodea are in the opposite direction of those observed between eusocial and solitary Hymenoptera (26). This disconnect, even among insect clades, points to the need for more taxonomically diverse analyses that can disentangle the role that life history, meiotic drive, and effective population size may have on the evolution of chromosome number.

Supplementary figures and tables

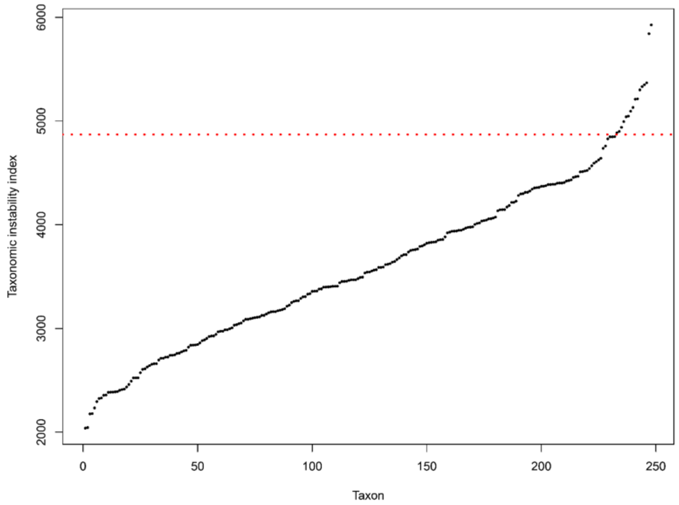

Figure S1 The red dotted line represents the cutoff point of 4780. Approximately 94% of the taxa fall below this cutoff point.

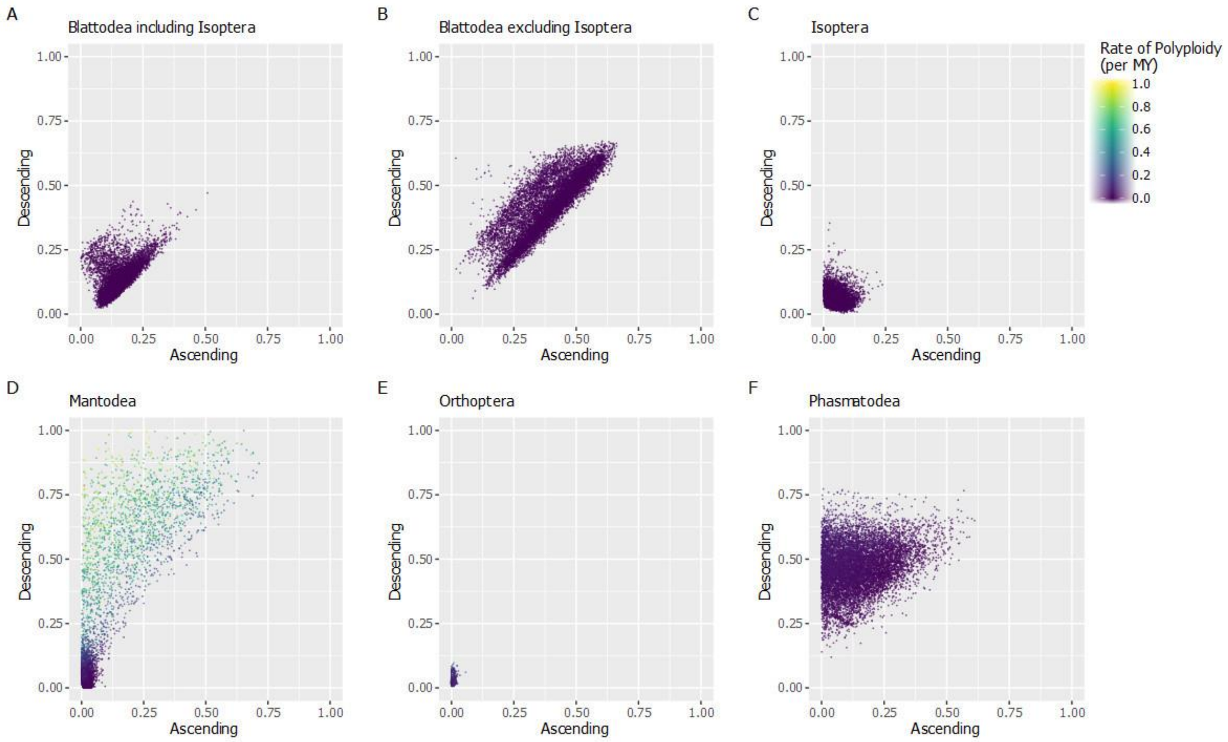

Figure S2 Rate of chromosome gain (ascending) and loss (descending) in five orders of Polyneoptera. Each point is coloured based on the inferred rate of polyploidy.

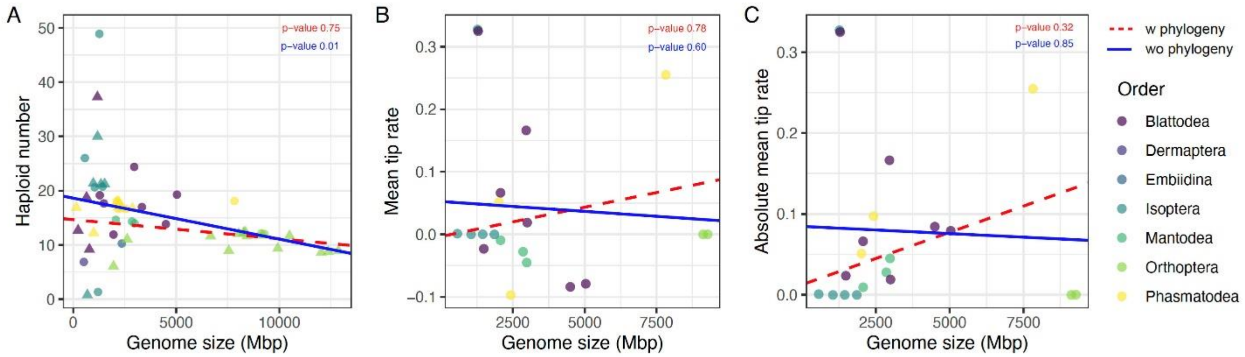

Figure S3 Impacts of genome size. Blue lines indicate regression line without phylogenetic correction and red dashed line indicates regression line with phylogenetic correction. P-values are printed in the same color in each plot. Circles are taxa that are present in the phylogeny and triangles are taxa that are not present in the phylogeny. A) Haploid chromosome number and genome size B) Mean tip rate and genome size C) Absolute tip rate and genome size.

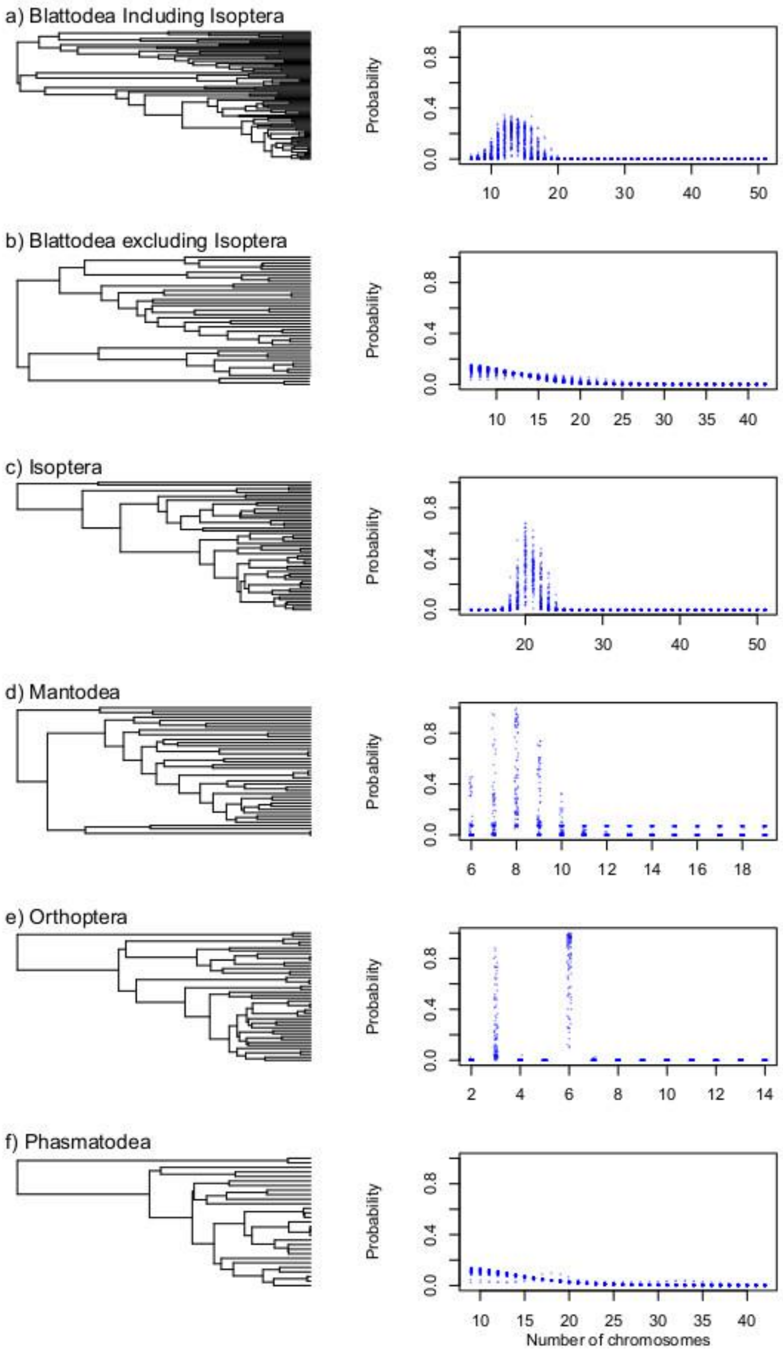

Figure S4 Ancestral state inference of chromosome number. Each point is the probability associated with a particular chromosome number on one of the 100 trees from the posterior distribution.

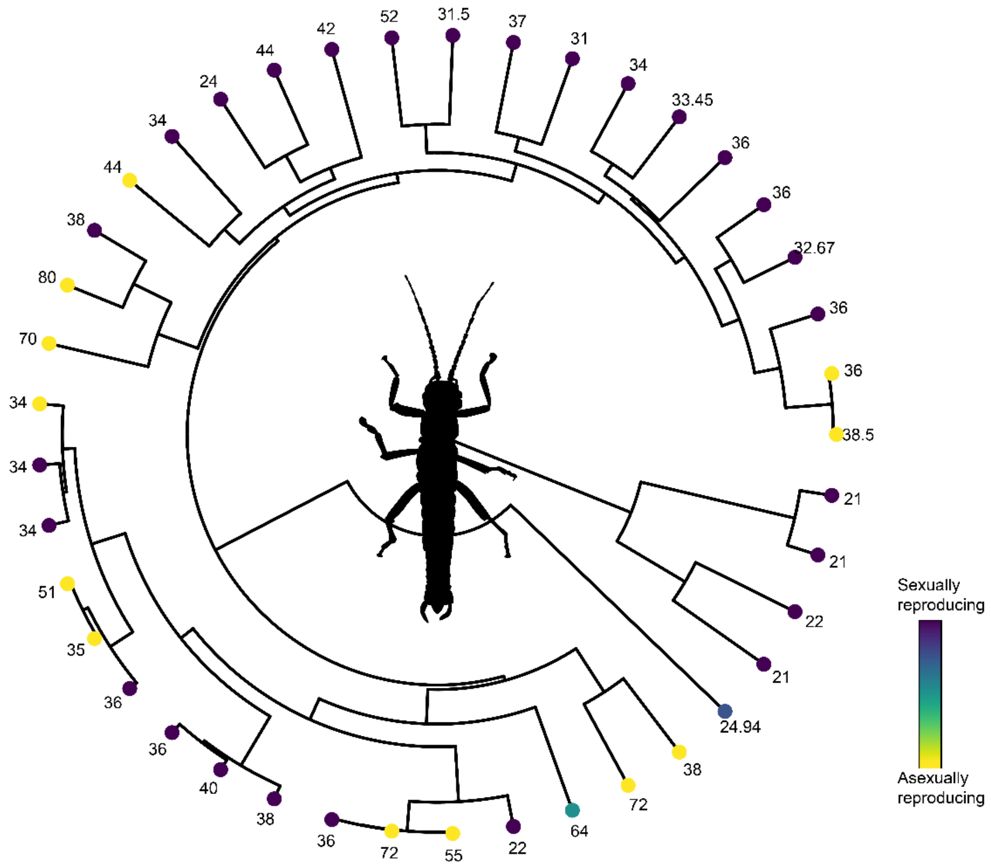

Figure S5 Phylogeny of Phasmatodea with reproductive mode and chromosome number. Tips are coloured according to the mode of reproduction (sexual or asexual). Some lineages show intermediate colours. These are lineages which have both sexual and asexual populations. The shade of colour indicates the probability of observing either reproductive modes in these lineages. The numbers indicate the mean chromosome number for each species.

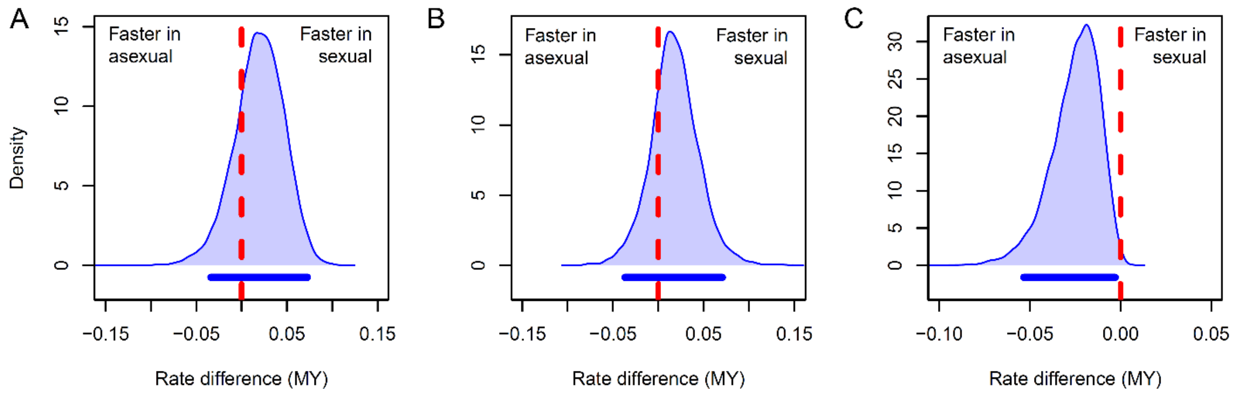

Figure S6 Rates of chromosome number evolution in sexual and asexual lineages in Phasmatodea. A) fission, B) fusion, and C) polyploidy. Bars below the plot indicates the 95% HPD interval.

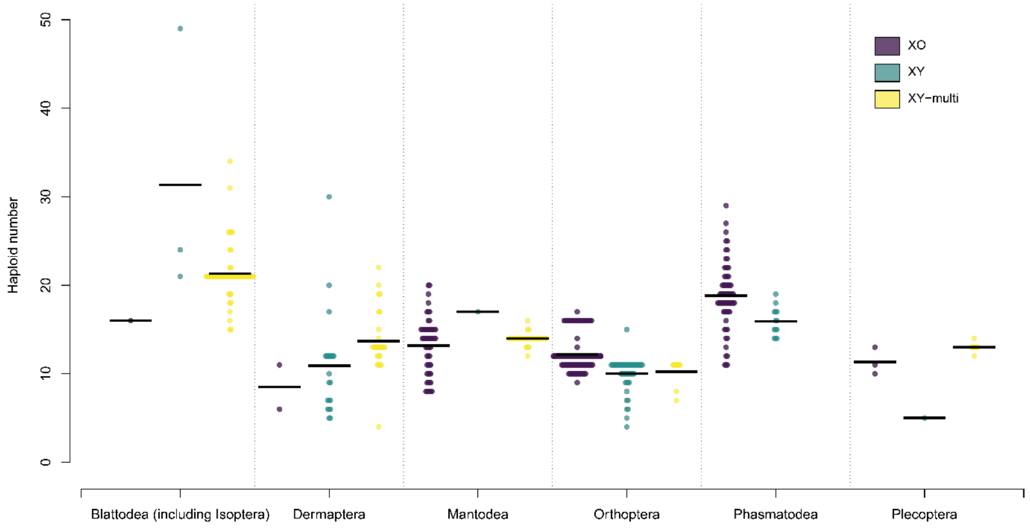

Figure S7 Variation in chromosome numbers parsed by sex chromosome system in six Polyneoptera orders. The vertical axis indicates the haploid chromosome count.

Table S1 Chromosome number, sex chromosome system and mode of reproduction of the studied taxa.

Order	family	genus	species	sex.system	SCS	scs.notes	fem.2n	mal.2n	hap
Blattodea	Blattellidae	Agmoblatta	Agmoblatta_thaxteri		XO		26	25	13
Blattodea	Blattellidae	Amazonina	Amazonina_conspersa		XO		24	23	12
Blattodea	Blattellidae	Amazonina	Amazonina_n.sp.		XO		26	25	13
Blattodea	Blattellidae	Amazonina	Amazonina_n.sp.38B		XO		26	25	13
Blattodea	Blaberidae	Archimandrita	Archimandrita_tessellata		XO		46	45	23
Blattodea	Polyphagidae	Arenivaga	Arenivaga_investigata		XO		46	45	23
Blattodea	Blattellidae	Attaphila	Attaphila_fungicola		XO		34	33	17
Blattodea	Blaberidae	Blaberus	Blaberus_atropos		XO		74	73	37
Blattodea	Blaberidae	Blaberus	Blaberus_cranifer		XO		74	73	37
Blattodea	Blaberidae	Blaberus	Blaberus_discoidalis		XO		38	37	19
Blattodea	Blaberidae	Blaberus	Blaberus_giganteus		XO		74	73	37
Blattodea	Blaberidae	Blaberus	Blaberus_parabolicus		XO		40	39	20
Blattodea	Blattidae	Blatta	Blatta_orientalis		XO		48	47	24
Blattodea	Blattellidae	Blattella	Blattella_bisignata		XO		24	23	12
Blattodea	Blattellidae	Blattella	Blattella_germanica		XO		24	23	12
Blattodea	Blattellidae	Blattella	Blattella_lituricollis_lituricollis		XO		26	25	13
Blattodea	Blattellidae	Blattella	Blattella_sauteri		XO		24	23	12
Blattodea	Blattellidae	Blattella	Blattella_sp.C43T		XO		28	27	14
Blattodea	Blattellidae	Blattella	Blattella_sp.D		XO		30	29	15
Blattodea	Blattellidae	Blattella	Blattella_sp.E71A		XO		50	49	25
Blattodea	Blattellidae	Blattella	Blattella_vaga		XO		24	23	12
Blattodea	Blaberidae	Byrsotria	Byrsotria_fumigata		XO		48	47	24
Blattodea	Blaberidae	Capucina	Capucina_patula		XO		38	37	19

Blattodea	Blattellidae	Cariblatta	Cariblatta_lutea_minima		XO		26	25	13
Blattodea	Polyphagidae	Cryptocercus	Cryptocercus_punctulatus	gonochorous	XO	multivalent absent order listed as Dictyoptera	48	47	24
Blattodea	Polyphagidae	Cryptocercus	Cryptocercus_primarius		XO	multivalent absent order listed as Dictyoptera	20	19	10
Blattodea	Polyphagidae	Cryptocercus	Cryptocercus_punctulatus		XO	multivalent absent order listed as Dictyoptera	38	37	19
Blattodea	Polyphagidae	Cryptocercus	Cryptocercus_relictus		XO	multivalent absent order listed as Dictyoptera	18	17	9
Blattodea	Blattidae	Cryptocercus	Cryptocercus_punctulatus		XO		40	39	20
Blattodea	Blattellidae	Dendroblatta	Dendroblatta_cnephaia		XO		32	31	16
Blattodea	Blattellidae	Dendroblatta	Dendroblatta_sobrina		XO		26	25	13
Blattodea	Blattidae	Deropeltis	Deropeltis_erythrocephala		XO		50	49	25
Blattodea	Blaberidae	Diploptera	Diploptera_punctata		XO		48	47	24
Blattodea	Blattidae	Duchailluia	Duchailluia_n.sp.		XO		42	41	21
Blattodea	Blattellidae	Ectobius	Ectobius_pallidus		XO		22	21	11
Blattodea	Blaberidae	Epilampra	Epilampra_abdomennigrum		XO		42	41	21
Blattodea	Blaberidae	Epilampra	Epilampra_grisea		XO		50	49	25
Blattodea	Blaberidae	Epilampra	Epilampra_maculicollis		XO		38	37	19
Blattodea	Blaberidae	Epilampra	Epilampra_maya		XO		36	35	18
Blattodea	Blaberidae	Epilampra	Epilampra_sagitta		XO		44	43	22
Blattodea	Blaberidae	Epilampra	Epilampra_sp.81B		XO		40	39	20
Blattodea	Blaberidae	Eublaberus	Eublaberus_distanti		XO		66	65	33
Blattodea	Blaberidae	Eublaberus	Eublaberus_posticus		XO		44	43	22
Blattodea	Blattidae	Eurycotis	Eurycotis_biolleyi		XO		22	21	11
Blattodea	Blattidae	Eurycotis	Eurycotis_decipiens		XO		28	27	14
Blattodea	Blattidae	Eurycotis	Eurycotis_floridana		XO		28	27	14
Blattodea	Blaberidae	Galiblatta	Galiblatta_williamsi		XO		28	27	14
Blattodea	Blattellidae	Gislenia	Gislenia_australica		XO		34	33	17
Blattodea	Blaberidae	Gromphadorhina	Gromphadorhina_brunneri		XO		64	63	32
Blattodea	Blaberidae	Gromphadorhina	Gromphadorhina_chopardi		XO		76	75	38
Blattodea	Blaberidae	Gromphadorhina	Gromphadorhina_portentosa		XO		64	63	32
Blattodea	Blattellidae	Hemithyrsocera	Hemithyrsocera_latera/is		XO		26	25	13

Blattodea	Blaberidae	Hormetica	Hormetica_scrobiculata	XO	32	31	16
Blattodea	Blaberidae	Hormetica	Hormetica_ventralis	XO	32	31	16
Blattodea	Blaberidae	Hyporhicnoda	Hyporhicnoda_litomorpha	XO	44	43	22
Blattodea	Blattellidae	Ischnoptera	Ischnoptera_castanea	XO	34	33	17
Blattodea	Blattellidae	Ischnoptera	Ischnoptera_deropeltiformis	XO	50	49	25
Blattodea	Blattellidae	Ischnoptera	Ischnoptera_galibi	XO	44	43	22
Blattodea	Blattellidae	Ischnoptera	Ischnoptera_mura	XO	34	33	17
Blattodea	Blattellidae	Ischnoptera	Ischnoptera_panamae	XO	40	39	20
Blattodea	Blattellidae	Ischnoptera	Ischnoptera_rufa_rufa	XO	40	39	20
Blattodea	Blattellidae	Ischnoptera	Ischnoptera_sp.93B	XO	38	37	19
Blattodea	Blattidae	Lamproblatta	Lamproblatta_albipalpus	XO	18	17	9
Blattodea	Blaberidae	Lanxoblatta	Lanxoblatta_emarginata	XO	66	65	33
Blattodea	Polyphagidae	Latindia	Latindia_sp.3		NA	22	11
Blattodea	Blaberidae	Leucophaea	Leucophaea_maderae	XO	24	23	12
Blattodea	Blattellidae	Loboptera	Loboptera_decipiens	XO	34	33	17
Blattodea	Blattellidae	Lobopterella	Lobopterella_dimidiatipes	XO	38	37	19
Blattodea	Blattellidae	Lophoblatta	Lophoblatta_brevis	XO	32	31	16
Blattodea	Blattellidae	Lophoblatta	Lophoblatta_fissa	XO	16	15	8
Blattodea	Blattellidae	Lupparia	Lupparia_notulata	XO	20	19	10
Blattodea	Blaberidae	Macropanesthia	Macropanesthia_rhinoceros	XO	80	79	40
Blattodea	Blattellidae	Margattea	Margattea_punctulata	XO	32	31	16
Blattodea	Blattellidae	Nahublattella	Nahublattella_n.sp.72B	XO	20	19	10
Blattodea	Blaberidae	Nauphoeta	Nauphoeta_cinerea	XO	38	37	19
Blattodea	Blaberidae	Panchlora	Panchlora_nivea	XO	36	35	18
Blattodea	Blaberidae	Panchlora	Panchlora_sp.	XO	32	31	16
Blattodea	Blaberidae	Panchlora	Panchlora_viridis	XO	38	37	19
Blattodea	Blaberidae	Panesthia	Panesthia_sp.	XO	38	37	19
Blattodea	Blaberidae	Panesthia	Panesthia_stellata	XO	38	37	19
Blattodea	Blattellidae	Parasymploce	Parasymploce_limbata	XO	24	23	12
Blattodea	Blattellidae	Parasymploce	Parasymploce_sp.25T	XO	32	31	16
Blattodea	Blattellidae	Parasymploce	Parasymploce_sp.30T	XO	32	31	16

Blattodea	Blattellidae	Parcoblatta	Parcoblatta_fulvescens	XO		38	37	19
Blattodea	Blattellidae	Parcoblatta	Parcoblatta_pennsylvanica	XO		38	37	19
Blattodea	Blattidae	Pelmatosilpha	Pelmatosilpha_coriacea	XO		38	37	19
Blattodea	Blattidae	Periplaneta	Periplaneta_americana	XO		34	33	17
Blattodea	Blattidae	Periplaneta	Periplaneta_australasiae	XO		28	27	14
Blattodea	Blattidae	Periplaneta	Periplaneta_brunnea	XO		28	27	14
Blattodea	Blattidae	Periplaneta	Periplaneta_fuliginosa	XO		28	27	14
Blattodea	Blattidae	Periplaneta	Periplaneta_japonica	XO		34	33	17
Blattodea	Blaberidae	Petasodes	Petasodes_dominicana	XO		54	53	27
Blattodea	Blaberidae	Phoetalia	Phoetalia_circumvagans	XO		54	53	27
Blattodea	Blaberidae	Phortioica	Phortioica_phoraspoides	XO		66	65	33
Blattodea	Blaberidae	Pinaconota	Pinaconota_sicca	XO		34	33	17
Blattodea	Polyphagidae	Polyphaga	Polyphaga_aegyptiaca	XO		64	63	32
Blattodea	Blaberidae	Proscratea	Proscratea_complanata	XO		36	35	18
Blattodea	Blattellidae	Pseudomops	Pseudomops_septentrionalis	XO		32	31	16
Blattodea	Blaberidae	Pycnoscelus	Pycnoscelus_indicus	XO		36	35	18
Blattodea	Blaberidae	Pycnoscelus	Pycnoscelus_surinamensis			35	NA	18
Blattodea	Blaberidae	Pycnoscelus	Pycnoscelus_surinamensis			53	NA	27
Blattodea	Blaberidae	Rhabdoblatta	Rhabdoblatta_annandalei	XO		50	49	25
Blattodea	Blaberidae	Rhabdoblatta	Rhabdoblatta_sp.39T	XO		32	31	16
Blattodea	Blattellidae	Shawella	Shawella_couloniana	XO		36	35	18
Blattodea	Blaberidae	Sp.	Sp._sp41B	XO		30	29	15
Blattodea	Blattellidae	Supella	Supella_longipalpa	XO		20	19	10
Blattodea	Blattellidae	Symploce	Symploce_capitata	XO		28	27	14
Blattodea	Blattellidae	Symploce	Symploce_fusca	XO		30	29	15
Blattodea	Blattellidae	Symplocodes	Symplocodes_marmorata	XO		40	39	20
Blattodea	Blattellidae	Symplocodes	Symplocodes_n.sp.50	XO		38	37	19
Blattodea	Blattellidae	Xestoblatta	Xestoblatta_immaculata	XO		38	37	19
Dermaptera	Forficulidae	Anechura	Anechura_bipunctata	XY		24	24	12
Dermaptera	Labiduridae	Anisolabis	Anisolabis_marginalis	complex XY	XXY	26	25	13
Dermaptera	Labiduridae	Anisolabis	Anisolabis_maritima	complex XY	XXY	26	25	13

Dermaptera	Labiduridae	Anisolabis	Anisolabis_sp	complex XY	XXXY	44	42	22
Dermaptera	Labiduridae	Anisolabis	Anisolabis_annuipes	complex XY		26	25	13
Dermaptera	Forficulidae	Apterygida	Apterygida_albipennis	XY		24	24	12
Dermaptera	Arixenidae	Arixenia	Arixenia_esau	XY		60	60	30
Dermaptera	Labiidae	Chaetospania	Chaetospania_brunner	complex XY	XXXXY	34	31	17
Dermaptera	Labiidae	Chaetospania	Chaetospania_sp.1	complex XY	XXY	22	21	11
Dermaptera	Labiidae	Chaetospania	Chaetospania_sp.2	complex XY	XXY	22	21	11
Dermaptera	Pygicranidae	Dacnodes	Dacnodes_shortridgei	XO		12	11	6
Dermaptera	Pygicranidae	Diplatys	Diplatys_gladiator	XY		18	18	9
Dermaptera	Labiduridae	Euborellia	Euborellia_annulipes	complex XY	XXY	26	25	13
Dermaptera	Labiduridae	Euborellia	Euborellia_moesta	complex XY	XXY	26	25	13
Dermaptera	Labiduridae	Euborellia	Euborellia_stali	complex XY	XXY	26	25	13
Dermaptera	Forficulidae	Forficula	Forficula_scudderi	XY		24	24	12
Dermaptera	Forficulidae	Forficula	Forficula_smyrnensis	complex XY	XXY	29	21	15
Dermaptera	Forficulidae	Forficula	Forficula_auricularia	complex XY	XXY	24	24	12
Dermaptera	Forficulidae	Forficula	Forficula_auricularia	complex XY	XXY	24	24	12
Dermaptera	Forficulidae	Forficula	Forficula_auricularia	complex XY	XXY	24	25	12
Dermaptera	Forficulidae	Forficula	Forficula_scudderi	XY		24	24	12
Dermaptera	Forficulidae	Forficula	Forficula_smyrnensis	complex XY	XXY	22	21	11
Dermaptera	Forficulidae	Forficula	Forficula_sp	complex XY	XXY	28	27	14
Dermaptera	Forficulidae	Forficula	Forficula_auricularia	complex XY	XXY	26	25	13
Dermaptera	Forficulidae	Forficula	Forficula_auricularia	complex XY	XXY	25	NA	13
Dermaptera	Forficulidae	Forficula	Forficula_auricularia	complex XY	XXY	26	NA	13
Dermaptera	Forficulidae	Forficula	Forficula_auricularia	XY	XY males (fused X1X2)	24	24	12
Dermaptera	Forficulidae	Forficula	Forficula_auricularia	XY		24	24	12
Dermaptera	Forficulidae	Forficula	Forficula_auricularia	complex XY	XXY	NA	25	13
Dermaptera	Forficulidae	Forficula	Forficula_auricularia			24	NA	12
Dermaptera	Labiduridae	Gonolabis	Gonolabis_brunneri	XY		24	24	12
Dermaptera	Hemimeridae	Hemimerus	Hemimerus_bouvieri	complex XY	XXY	8	7	4
Dermaptera	Labiidae	Labia	Labia_minor	XY		14	14	7
Dermaptera	Labiidae	Labia	Labia_sp.	XY		18	18	9

Dermaptera	Labiduridae	Labidura	Labidura_riparia		XY	heterochromatic Y from Brazil	12	12	6
Dermaptera	Labiduridae	Labidura	Labidura_truncata		XY		10	10	5
Dermaptera	Labiduridae	Labidura	Labidura_bidens		XY		12	12	6
Dermaptera	Labiduridae	Labidura	Labidura_riparia		XY		14	14	7
Dermaptera	Labiduridae	Labidura	Labidura_truncata		XY		10	10	5
Dermaptera	Labiduridae	Labidura	Labidura_riparia		XY		12	12	6
Dermaptera	Labiduridae	Labidura	Labidura_bengalensis		XY	from India	14	14	7
Dermaptera	Labiduridae	Nala	Nala_lividipes		complex XY	XXY	38	37	19
Dermaptera	Labiduridae	Nala	Nala_lividipes		XY		34	34	17
Dermaptera	Labiduridae	Nala	Nala_lividipes		XY		40	40	20
Dermaptera	Labiidae	Nesogaster	Nesogaster_erichsoni		complex XY	XXY	22	21	11
Dermaptera	Labiidae	Nesogaster	Nesogaster_halli		XO		22	19	11
Dermaptera	Labiduridae	Notolabis	Notolabis_occidentalis		XY		24	24	12
Dermaptera	Labiduridae	Parisopsalis	Parisopsalis_spryi		complex XY	XXXY	34	32	17
Dermaptera	Labiidae	Prolabia	Prolabia_arachidis		complex XY	XXXY	40	38	20
Dermaptera	Forficulidae	Pseudochelidura	Pseudochelidura_sinuata		complex XY	XXY	26	25	13
Dermaptera	Labiidae	unknown	unknown_Sp.		complex XY	XXY	38	37	19
Dermaptera	Pygicranidae	unknown	unknown_sp.		XY		20	20	10
Embiidina	Embiidae	Cleomia	Cleomia_guareschii		XO		22	21	11
Embiidina	Embiidae	Embia	Embia_nuragica		XO		24	23	12
Embiidina	Embiidae	Embia	Embia_ramburi		XO		22	21	11
Embiidina	Embiidae	Embia	Embia_tyrrhenica		XO		22	21	11
Embiidina	Oligotomidae	Haploembia	Haploembia_palaui		XO		22	21	11
Embiidina	Oligotomidae	Haploembia	Haploembia_solieri		XO		20	19	10
Embiidina	Oligotomidae	Oligotoma	Oligotoma_japonica		XO		20	19	10
Embiidina	Oligotomidae	Oligotoma	Oligotoma_saundersi		XO		22	21	11
Grylloblata	Grylloblatidae	Galloisiana	Galloisiana_nipponensis	gonochorous	XY	heteromorphic	30	30	15
Grylloblata	Grylloblatidae	Grylloblatta	Grylloblatta_campodeioformis	gonochorous	XY	metacentric X and Y associated by one arm	30	NA	15
Isoptera	Termitidae	Acidnotermes	Acidnotermes_praus	gonochorous	complex XY|homomorphic	X1X1X2X2/X1X2Y1Y2 sex-linked multivalent in males	42	NA	21

Isoptera	Termitidae	Afrosubulitermes	Afrosubulitermes_congoensis	gonochorous	complex XY|homomorphic	X1X1X2X2/X1X2Y1Y2 sex-linked multivalent in males	42	NA	21
Isoptera	Termitidae	Amitermes	Amitermes_darwini	gonochorous	complex XY|homomorphic	X1X1X2X2/X1X2Y1Y2 sex-linked multivalent in males	42	42	21
Isoptera	Termitidae	Amitermes	Amitermes_eucalipti				42	NA	21
Isoptera	Termitidae	Amitermes	Amitermes_germanus				42	NA	21
Isoptera	Termitidae	Amitermes	Amitermes_parvus	gonochorous	complex XY|homomorphic	X1X1X2X2/X1X2Y1Y2 sex-linked multivalent in males	42	42	21
Isoptera	Kalotermitidae	Bifiditermes	Bifiditermes_improbus	gonochorous	complex XY|homomorphic	X1X1X2X2/X1X2Y1	36	35	18
Isoptera	Rhinotermitidae	Coptotermes	Coptotermes_acinaciformis	gonochorous	complex XY|homomorphic	X1X1X2X2/X1X2Y1Y2 sex-linked multivalent in males	42	42	21
Isoptera	Termitidae	Crenetermes	Crenetermes_albotarsalis	gonochorous	complex XY|homomorphic	X1X1X2X2/X1X2Y1Y2 sex-linked multivalent in males	42	NA	21
Isoptera	Kalotermitidae	Cryptotermes	Cryptotermes_domesticus	gonochorous	complex XY|homomorphic	X1X1X2X2/X1X2Y1	30	29	15
Isoptera	Kalotermitidae	Cryptotermes	Cryptotermes_secundus				40	NA	20
Isoptera	Kalotermitidae	Cryptotermes	Cryptotermes_austrinus	gonochorous			NA	40	20
Isoptera	Kalotermitidae	Cryptotermes	Cryptotermes_brevis	gonochorous	complex XY|homomorphic	X1X1/X1Y1Y2	36	37	18
Isoptera	Kalotermitidae	Cryptotermes	Cryptotermes_cristatus	gonochorous			NA	46	23
Isoptera	Kalotermitidae	Cryptotermes	Cryptotermes_cynocephalus	gonochorous	complex XY|homomorphic	X1X1X2X2/X1X2Y1	44	43	22
Isoptera	Kalotermitidae	Cryptotermes	Cryptotermes_domesticus	gonochorous	complex XY|homomorphic	X1X1X2X2/X1X2Y1	30	29	15
Isoptera	Kalotermitidae	Cryptotermes	Cryptotermes_dudleyi	gonochorous			40	40	20
Isoptera	Kalotermitidae	Cryptotermes	Cryptotermes_gearyi	gonochorous	XY|homomorphic		NA	48	24
Isoptera	Kalotermitidae	Cryptotermes	Cryptotermes_papulosus	gonochorous			42	NA	21
Isoptera	Kalotermitidae	Cryptotermes	Cryptotermes_primus	gonochorous			30	NA	15
Isoptera	Kalotermitidae	Cryptotermes	Cryptotermes_queenslandis	gonochorous	complex XY|homomorphic	X1X1X2X2/X1X2Y1	42	41	21
Isoptera	Kalotermitidae	Cryptotermes	Cryptotermes_riverinae	gonochorous			42	42	21
Isoptera	Kalotermitidae	Cryptotermes	Cryptotermes_secundus	gonochorous			40	40	20
Isoptera	Kalotermitidae	Cryptotermes	Cryptotermes_sp.	gonochorous			NA	47	24

Isoptera	Termitidae	Cubitermes	Cubitermes_exiguus	gonochorous	complex XY|homomorphic	X1X1X2X2/X1X2Y1Y2 sex-linked multivalent in males	42	NA	21
Isoptera	Termitidae	Cubitermes	Cubitermes_sankurensis	gonochorous	complex XY|homomorphic	X1X1X2X2/X1X2Y1Y2 sex-linked multivalent in males	42	NA	21
Isoptera	Termitidae	Cubitermes	Cubitermes_sp._1.	gonochorous	complex XY|homomorphic	X1X1X2X2/X1X2Y1Y2 sex-linked multivalent in males	42	NA	21
Isoptera	Termitidae	Cubitermes	Cubitermes_weissi	gonochorous	complex XY|homomorphic	X1X1X2X2/X1X2Y1Y2 sex-linked multivalent in males	42	NA	21
Isoptera	Termitidae	Drepanotermes	Drepanotermes_septentrionalis	gonochorous	complex XY|homomorphic	X1X1X2X2/X1X2Y1Y2 sex-linked multivalent in males	42	42	21
Isoptera	Termitidae	Ephelotermes	Ephelotermes_melachoma	gonochorous	complex XY|homomorphic	X1X1X2X2/X1X2Y1Y2 sex-linked multivalent in males	42	42	21
Isoptera	Termitidae	Ephelotermes	Ephelotermes_taylori	gonochorous	complex XY|homomorphic	X1X1X2X2/X1X2Y1Y2 sex-linked multivalent in males	42	42	21
Isoptera	Kalotermitidae	Glyptotermes	Glyptotermes_brevicornis	gonochorous			42	NA	21
Isoptera	Kalotermitidae	Glyptotermes	Glyptotermes_iridipennis	gonochorous			NA	42	21
Isoptera	Kalotermitidae	Incisitermes	Incisitermes_barretti	gonochorous			NA	42	21
Isoptera	Kalotermitidae	Incisitermes	Incisitermes_sp.	gonochorous			42	NA	21
Isoptera	Kalotermitidae	Kalotermes	Kalotermes_flavicollis		complex XY|homomorphic	X1X1X2X2/X1X2Y1	68	67	34
Isoptera	Kalotermitidae	Kalotermes	Kalotermes_convexus	gonochorous			NA	46	23
Isoptera	Termitidae	Kalotermes	Kalotermes_approximatus	gonochorous	complex XY|homomorphic	X1X1X2X2/X1X2Y1 sex-linked multivalent in males	34	33	17
Isoptera	Termitidae	Kalotermes	Kalotermes_approximatus	gonochorous	complex XY|homomorphic	X1X1X2X2/X1X2Y1Y2 sex-linked multivalent in males	32	NA	16
Isoptera	Termitidae	Kalotermes	Kalotermes_Flavicollis	gonochorous	complex XY|homomorphic	X1X1X2X2/X1X2Y1Y2 sex-linked multivalent in males	61	NA	31
Isoptera	Termitidae	Lophotermes	Lophotermes_septentrionalis	gonochorous	complex XY|homomorphic	X1X1X2X2/X1X2Y1Y2 sex-linked multivalent in males	42	42	21
Isoptera	Termitidae	Macrognathotermes	Macrognathotermes_sunteri	gonochorous	complex XY|homomorphic	X1X1X2X2/X1X2Y1Y2 sex-linked multivalent in males	42	42	21

Isoptera	Termitidae	Macrotermes	Macrotermes_bellicosus	gonochorous	complex XY|homomorphic	X1X1X2X2/X1X2Y1Y2 sex-linked multivalent in males	42	NA	21
Isoptera	Termitidae	Mastotermes	Mastotermes_darwiniensis	gonochorous	XY|homomorphic	multivalent absent	98	NA	49
Isoptera	Termitidae	Microcerotermes	Microcerotermes_boreus	gonochorous	complex XY|homomorphic	X1X1X2X2/X1X2Y1Y2 sex-linked multivalent in males	42	42	21
Isoptera	Termitidae	Microcerotermes	Microcerotermes_nervosus	gonochorous	complex XY|homomorphic	X1X1X2X2/X1X2Y1Y2 sex-linked multivalent in males	42	42	21
Isoptera	Termitidae	Microcerotermes	Microcerotermes_fuscotibiatis	gonochorous	complex XY|homomorphic	X1X1X2X2/X1X2Y1Y2 sex-linked multivalent in males	42	NA	21
Isoptera	Termitidae	Microcerotermes	Microcerotermes_parvulus	gonochorous	complex XY|homomorphic	X1X1X2X2/X1X2Y1Y2 sex-linked multivalent in males	42	NA	21
Isoptera	Termitidae	Microcerotermes	Microcerotermes_sp.1	gonochorous	complex XY|homomorphic	X1X1X2X2/X1X2Y1Y2 sex-linked multivalent in males	44	NA	22
Isoptera	Termitidae	Microcerotermes	Microcerotermes_sp.2	gonochorous	complex XY|homomorphic	X1X1X2X2/X1X2Y1Y2 sex-linked multivalent in males	42	NA	21
Isoptera	Termitidae	Microtermes	Microtermes_sp.1	gonochorous	complex XY|homomorphic	X1X1X2X2/X1X2Y1Y2 sex-linked multivalent in males	42	NA	21
Isoptera	Termitidae	Nasutitermes	Nasutitermes_graveolus	gonochorous	complex XY|homomorphic	X1X1X2X2/X1X2Y1Y2 sex-linked multivalent in males	42	42	21
Isoptera	Termitidae	Nasutitermes	Nasutitermes_longipennis	gonochorous	complex XY|homomorphic	X1X1X2X2/X1X2Y1Y2 sex-linked multivalent in males	42	42	21
Isoptera	Termitidae	Nasutitermes	Nasutitermes_triodiae	gonochorous	complex XY|homomorphic	X1X1X2X2/X1X2Y1Y2 sex-linked multivalent in males	42	42	21
Isoptera	Termitidae	Nasutitermes	Nasutitermes_arboreus	gonochorous	complex XY|homomorphic	X1X1X2X2/X1X2Y1Y2 sex-linked multivalent in males	42	NA	21
Isoptera	Kalotermitidae	Neotermes	Neotermes_insularis	gonochorous	complex XY	XXXYYY	52	52	26
Isoptera	Kalotermitidae	Neotermes	Neotermes_insularis	gonochorous			NA	60	30
Isoptera	Termitidae	Noditermes	Noditermes_lamanianus	gonochorous	complex XY|homomorphic	X1X1X2X2/X1X2Y1Y2 sex-linked multivalent in males	38	NA	19
Isoptera	Termitidae	Odontotermes	Odontotermes_redemanni	gonochorous	complex XY|homomorphic	X1X1X2X2/X1X2Y1Y2 sex-linked multivalent in males	48	NA	24

Isoptera	Termitidae	Odontotermes	Odontotermes_snyderi	gonochorous	complex XY|homomorphic	X1X1X2X2/X1X2Y1Y2 sex-linked multivalent in males	42	NA	21
Isoptera	Termitidae	Odontotermes	Odontotermes_sp.1	gonochorous	complex XY|homomorphic	X1X1X2X2/X1X2Y1Y2 sex-linked multivalent in males	42	NA	21
Isoptera	Termitidae	Ophiotermes	Ophiotermes_mandibularis	gonochorous	complex XY|homomorphic	X1X1X2X2/X1X2Y1Y2 sex-linked multivalent in males	42	NA	21
Isoptera	Termitidae	Pericapritermes	Pericapritermes_sp.1	gonochorous	complex XY|homomorphic	X1X1X2X2/X1X2Y1Y2 sex-linked multivalent in males	42	NA	21
Isoptera	Stolotermitidae	Porotermes	Porotermes_adamsoni	gonochorous			40	40	20
Isoptera	Kalotermitidae	Procryptotermes	Procryptotermes_australiensis	gonochorous	complex XY	X1X1X2X2/X1X2Y1	48	47	24
Isoptera	Termitidae	Procubitermes	Procubitermes_sp._1	gonochorous	complex XY|homomorphic	X1X1X2X2/X1X2Y1Y2 sex-linked multivalent in males	38	NA	19
Isoptera	Termitidae	Protermes	Protermes_minimus	gonochorous	complex XY|homomorphic	X1X1X2X2/X1X2Y1Y2 sex-linked multivalent in males	42	NA	21
Isoptera	Termitidae	Pseudacanthotermes	Pseudacanthotermes_militaris	gonochorous	complex XY|homomorphic	X1X1X2X2/X1X2Y1Y2 sex-linked multivalent in males	42	NA	21
Isoptera	Rhinotermitidae	Reticulitermes	Reticulitermes_urbis	gonochorous	homomorphic		42	42	21
Isoptera	Rhinotermitidae	Reticulitermes	Reticulitermes_lucifugus_grassei	gonochorous	complex XY|homomorphic	X1X1X2X2/X1X2Y1Y2 sex-linked multivalent in males	42	42	21
Isoptera	Termitidae	Reticulitermes	Reticulitermes_flavipes	gonochorous	complex XY|homomorphic	X1X1X2X2/X1X2Y1Y2 sex-linked multivalent in males	42	NA	21
Isoptera	Termitidae	Reticulitermes	Reticulitermes_lucifugus	gonochorous	complex XY|homomorphic	X1X1X2X2/X1X2Y1Y2 sex-linked multivalent in males	42	NA	21
Isoptera	Termitidae	Reticutitermes	Reticutitermes_santonensis	gonochorous	complex XY|homomorphic	X1X1X2X2/X1X2Y1Y2 sex-linked multivalent in males	42	NA	21
Isoptera	Termitidae	Schedorhinotermes	Schedorhinotermes_lamanianus	gonochorous	complex XY|homomorphic	X1X1X2X2/X1X2Y1Y2 sex-linked multivalent in males	38	NA	19
Isoptera	Stolotermitidae	Stolotermes	Stolotermes_victoriensis	gonochorous	XO		32	31	16
Isoptera	Termitidae	Tenuirostritermes	Tenuirostritermes_tenuirostris	gonochorous	complex XY|homomorphic	X1X1X2X2/X1X2Y1Y2 sex-linked multivalent in males	NA	NA	

Isoptera	Termitidae	Thoracotermes	Thoracotermes_macrothorax	gonochorous	complex XY|homomorphic	X1X1X2X2/X1X2Y1Y2 sex-linked multivalent in males	42	NA	21
Isoptera	Termitidae	Tuberculitermes	Tuberculitermes_bycanistes	gonochorous	complex XY|homomorphic	X1X1X2X2/X1X2Y1Y2 sex-linked multivalent in males	42	NA	21
Isoptera	Termitidae	Tumulitermes	Tumulitermes_pastinator	gonochorous	complex XY|homomorphic	X1X1X2X2/X1X2Y1Y2 sex-linked multivalent in males	42	42	21
Isoptera	Termitidae	Unguitermes	Unguitermes_bouilloni	gonochorous	complex XY|homomorphic	X1X1X2X2/X1X2Y1Y2 sex-linked multivalent in males	42	NA	21
Isoptera	Termitidae	Zootermopsis	Zootermopsis_angusticollis	gonochorous	complex XY|homomorphic	X1X1X2X2/X1X2Y1Y2 sex-linked multivalent in males	52	NA	26
Isoptera	Termitidae	Zootermopsis	Zootermopsis_angusticollis	gonochorous	complex XY|homomorphic	X1X1X2X2/X1X2Y1Y2 sex-linked multivalent in males	52	NA	26
Isoptera	Termitidae	Zootermopsis	Zootermopsis_nevadensis	gonochorous	complex XY|homomorphic	X1X1X2X2/X1X2Y1Y2 sex-linked multivalent in males	52	NA	26
Mantodea	Hymenopodidae	Acanthops	Acanthops_falcata		XO		20	19	10
Mantodea	Hymenopodidae	Acanthops	Acanthops_godmani		XO		20	19	10
Mantodea	Hymenopodidae	Acontiothespis	Acontiothespis_cordillerae_vitrea		XO		16	15	8
Mantodea	Hymenopodidae	Acontiothespis	Acontiothespis_multicolor				16	NA	8
Mantodea	Hymenopodidae	Acontiothespis	Acontiothespis_sp.		XO		16	15	8
Mantodea	Mantidae	Aethalochroa	Aethalochroa_ashmoliana		XO		30	29	15
Mantodea	Mantidae	Ameles	Ameles_abjecta		XO		30	29	15
Mantodea	Mantidae	Ameles	Ameles_heldreichi		XO		29	28	15
Mantodea	Mantidae	Ameles	Ameles_sp.		XO		21	20	11
Mantodea	Amorphoscelidae	Amorphoscelis	Amorphoscelis_indica		XO		34	33	17
Mantodea	Mantidae	Angela	Angela_guianensis				NA	19	10
Mantodea	Hymenopodidae	Antemna	Antemna_rapax		complex XY	XXY	28	27	14
Mantodea	Mantidae	Antistia	Antistia_sp.		XO		28	27	14
Mantodea	Mantidae	Apterornantis	Apterornantis_bolivari		XO		30	29	15
Mantodea	Mantidae	Archimantis	Archimantis_quinquelobata		complex XY		28	27	14
Mantodea	Mantidae	Archimantis	Archimantis_sobrina		complex XY		28	27	14
Mantodea	Mantidae	Arneles	Arneles_cypria		XO		27	26	14

Mantodea	Mantidae	Bisanthe	Bisanthe_pulchripennis	XO		22	21	11
Mantodea	Mantidae	Bolbe	Bolbe_nigra	XO		30	25	15
Mantodea	Mantidae	Bolbe	Bolbe_pallida	XO		30	25	15
Mantodea	Mantidae	Brunneria	Brunneria_borealis			28	NA	14
Mantodea	Mantidae	Callimantis	Callimantis_antillarum	XO		18	17	9
Mantodea	Mantidae	Cheddikulama	Cheddikulama_straminea	complex XY	XXY	28	27	14
Mantodea	Mantidae	Choeradodis	Choeradodis_rhombicollis	complex XY	XXY	32	31	16
Mantodea	Amorphoscelidae	Cliomantis	Cliomantis_cornuta	XO		26	25	13
Mantodea	Mantidae	Compsothespis	Compsothespis_anomala	complex XY	XXY	30	23	15
Mantodea	Mantidae	Compsothespis	Compsothespis_natalica	complex XY	XXY	30	23	15
Mantodea	Hymenopodidae	Creobroter	Creobroter_gemmatus	XO		28	27	14
Mantodea	Hymenopodidae	Creobroter	Creobroter_laevicollis	XO		28	27	14
Mantodea	Hymenopodidae	Creobroter	Creobroter_urbanus	XO		28	27	14
Mantodea	Mantidae	Deiphobe	Deiphobe_brunneri	XO		20	19	10
Mantodea	Mantidae	Deiphobe	Deiphobe_indica	complex XY	XXY	28	27	14
Mantodea	Eremiaphilidae	Didymocorypha	Didymocorypha_lanceolata	XO		16	15	8
Mantodea	Eremiaphilidae	Didymocorypha	Didymocorypha_lanceolata	XO		18	17	9
Mantodea	Mantidae	Dystacta	Dystacta_alticeps	XO		26	25	13
Mantodea	Empusidae	Empusa	Empusa_egena	XO		28	27	14
Mantodea	Empusidae	Empusa	Empusa_pauperata	XO		28	27	14
Mantodea	Empusidae	Empusa	Empusa_spinosa	XO		28	27	14
Mantodea	Hymenopodidae	Euantissa	Euantissa_ornata	XO		34	33	17
Mantodea	Amorphoscelidae	Glabromantis	Glabromantis_nebulosa	XO		26	25	13
Mantodea	Empusidae	Gongylus	Gongylus_gongyloides	XO		28	27	14
Mantodea	Mantidae	Haldwania	Haldwania_liliputana	XO		16	15	8
Mantodea	Hymenopodidae	Harpagomantis	Harpagomantis_tricolor	XO		26	25	13
Mantodea	Hymenopodidae	Hestiasula	Hestiasula_brunneriana	XO		28	27	14
Mantodea	Mantidae	Hierodula	Hierodula_coarctata	complex XY		28	27	14
Mantodea	Mantidae	Hierodula	Hierodula_patellifera	complex XY	XXY	28	27	14
Mantodea	Mantidae	Hierodula	Hierodula_sp.	complex XY	XXY	28	27	14
Mantodea	Mantidae	Hierodula	Hierodula_tenuidentata	complex XY		28	27	14

Mantodea	Mantidae	Hierodula	Hierodula_venosa	complex XY	XXY		28	27	14
Mantodea	Mantidae	Hierodula	Hierodula_ventralis	complex XY			28	27	14
Mantodea	Mantidae	Holaptilon	Holaptilon_pusillulurn	XO			30	29	15
Mantodea	Mantidae	Hoplocorypha	Hoplocorypha_macra	XO			36	35	18
Mantodea	Mantidae	Hoplocorypha	Hoplocorypha_sp.	XO			28	27	14
Mantodea	Eremiaphilidae	Humbertiella	Humbertiella_indica	XO			24	23	12
Mantodea	Eremiaphilidae	Humbertiella	Humbertiella_n.sp.	XO			40	39	20
Mantodea	Eremiaphilidae	Humbertiella	Humbertiella_similis	XO			32	31	16
Mantodea	Eremiaphilidae	Humbertiella	Humbertiella_sp.	XO			22	21	11
Mantodea	Mantidae	Ima	Ima_fusca	XY			34	34	17
Mantodea	Mantidae	Iris	Iris_aratoria	XO			26	25	13
Mantodea	Mantidae	Kongobatha	Kongobatha_diadernata	XO			30	25	15
Mantodea	Mantidae	Leptomantis	Leptomantis_parva	XO			40	39	20
Mantodea	Mantidae	Ligaria	Ligaria_quadripunctata	XO			24	23	12
Mantodea	Mantidae	Liturgusa	Liturgusa_actuosa	XO			24	23	12
Mantodea	Mantidae	Liturgusa	Liturgusa_cursor	XO			34	33	17
Mantodea	Mantidae	Liturgusa	Liturgusa_maya	XO			18	17	9
Mantodea	Mantidae	Liturgusa	Liturgusa_sp.	XO			22	21	11
Mantodea	Mantidae	Mantis	Mantis_octospilota	complex XY	XXY		28	27	14
Mantodea	Mantidae	Mantis	Mantis_religiosa	complex XY	XXY		28	27	14
Mantodea	Mantoididae	Mantoida	Mantoida_schraderi	XO			38	37	19
Mantodea	Mantidae	Melliera	Melliera_brevipes	complex XY	XXY		28	27	14
Mantodea	Mantidae	Miomantis	Miomantis_sp.	XO			16	15	8
Mantodea	Mantidae	Nullabora	Nullabora_flavoguttata	complex XY			28	27	14
Mantodea	Mantidae	Oligonyx	Oligonyx_dohrnianus	XO			30	19	15
Mantodea	Mantidae	Orthodera	Orthodera_gunnii	complex XY	XXY		26	25	13
Mantodea	Mantidae	Orthodera	Orthodera_ministralis	complex XY	XXY		26	25	13
Mantodea	Mantidae	Orthoderina	Orthoderina_straminea	complex XY	XXY		26	25	13
Mantodea	Mantidae	Oxyopsis	Oxyopsis_rubicunda	complex XY	XXY		28	27	14
Mantodea	Mantidae	Paratenodera	Paratenodera_sinensis	complex XY	XXY		28	27	14
Mantodea	Mantidae	Parathespis	Parathespis_humbertiana	XO			32	31	16

Mantodea	Mantidae	Phyllovates	Phyllovates_tripunctata	complex XY	XXY	28	27	14
Mantodea	Mantidae	Polyspilota	Polyspilota_aeruginosa	complex XY	XXY	28	27	14
Mantodea	Mantidae	Polyspilota	Polyspilota_sp.	complex XY	XXY	28	27	14
Mantodea	Mantidae	Promiopteryx	Promiopteryx_granadensis	XO		20	19	10
Mantodea	Hymenopodidae	Pseudacanthops	Pseudacanthops_medusa			20	NA	10
Mantodea	Mantidae	Pseudomiopteryx	Pseudomiopteryx_infuscata	XO		18	17	9
Mantodea	Mantidae	Rhodamantis	Rhodamantis_pulchella	complex XY		28	27	14
Mantodea	Mantidae	Rhodamantis	Rhodamantis_sp3'	complex XY	XXY	28	27	14
Mantodea	Mantidae	Schizocephala	Schizocephala_bicornis	XO		30	27	15
Mantodea	Mantidae	Sp.	Sp._Sp	XO		30	25	15
Mantodea	Mantidae	Sphodromantis	Sphodromantis_gastrica	complex XY	XXY	28	27	14
Mantodea	Mantidae	Sphodropoda	Sphodropoda_sp4	complex XY	XXY	28	27	14
Mantodea	Mantidae	Sphodropoda	Sphodropoda_tristis	complex XY		28	27	14
Mantodea	Mantidae	Sphodrornantis	Sphodrornantis_viridis	complex XY	XXY	24	23	12
Mantodea	Mantidae	Stagomantis	Stagomantis_heterogamia	complex XY		28	27	14
Mantodea	Mantidae	Stagornantis	Stagornantis_carolina	complex XY		28	27	14
Mantodea	Mantidae	Stagrnatoptera	Stagrnatoptera_septentrionalis	complex XY		28	27	14
Mantodea	Mantidae	Statilia	Statilia_maculata	complex XY	XXY	28	27	14
Mantodea	Mantidae	Tenodera	Tenodera_aridifolia	complex XY	XXY	28	27	14
Mantodea	Mantidae	Tenodera	Tenodera_australasiae	complex XY	XXY	28	27	14
Mantodea	Mantidae	Tenodera	Tenodera_superstitiosa	complex XY	XXY	28	27	14
Mantodea	Mantidae	Thesprotia	Thesprotia_filum	XO		30	23	15
Mantodea	Mantidae	Thesprotia	Thesprotia_graminis	XO		30	23	15
Mantodea	Hymenopodidae	Tithrone	Tithrone_roseipennis	XO		16	15	8
Mantodea	Mantidae	Toxomantis	Toxomantis_sinensis	XO		28	27	14
Mantodea	Mantidae	Vates	Vates_pectinicornis	complex XY	XXY	28	27	14
Mantophasmatodea	Austrophasmatidae	Karoophasma	Karoophasma_biedouwense	XO		14	13	7
Orthoptera	Acrididae	Abracris	Abracris_dilecta	XO		24	23	12
Orthoptera	Acrididae	Abracris	Abracris_sp._B	XO		22	21	11
Orthoptera	Acrididae	Adimantus	Adimantus_cubiceps	XO		24	23	12
Orthoptera	Acrididae	Adimantus	Adimantus_ornatissimus	XO		24	23	12

Orthoptera	Tettigoniidae	Aerotegmina	Aerotegmina_kilimandjarica	gonochorous	XO		34	33	17
Orthoptera	Tettigoniidae	Aerotegmina	Aerotegmina_shengenae	gonochorous	XO		28	27	14
Orthoptera	Acrididae	Albretchia	Albretchia_palpata		XO		24	23	12
Orthoptera	Romaleidae	Alcamenes	Alcamenes_clarazianus		XO		24	23	12
Orthoptera	Acrididae	Aleuas	Aleuas_gracilis		XY		20	20	10
Orthoptera	Acrididae	Aleuas	Aleuas_lineatus		XY		20	20	10
Orthoptera	Acrididae	Aleuas	Aleuas_sp._1		XY		20	20	10
Orthoptera	Acrididae	Aleuas	Aleuas_sp._2		XY		22	22	11
Orthoptera	Acrididae	Aleuas	Aleuas_sp._3		XY		20	20	10
Orthoptera	Acrididae	Aleuas	Aleuas_vitticollis		XO		20	19	10
Orthoptera	Acrididae	Allotruxalis	Allotruxalis_sp.		XO		24	23	12
Orthoptera	Acrididae	Allotruxalis	Allotruxalis_strigata		XO		24	23	12
Orthoptera	Acrididae	Amblytropidia	Amblytropidia_australis		XO		24	23	12
Orthoptera	Romaleidae	Antandrus	Antandrus_viridus		XO		24	23	12
Orthoptera	Acrididae	Apacris	Apacris_rubrithorax		XO		24	23	12
Orthoptera	Acrididae	Apacris	Apacris_sp._1		XO		24	23	12
Orthoptera	Acrididae	Apolobamba	Apolobamba_prope_pulchra		XO		24	23	12
Orthoptera	Tristiridae	Atacamacris	Atacamacris_diminuta		XY		10	10	5
Orthoptera	Acrididae	Atrachelacris	Atrachelacris_olivaceus		XY		22	22	11
Orthoptera	Acrididae	Atrachelacris	Atrachelacris_unicolor		XY		22	22	11
Orthoptera	Ommexechidae	Aucacris	Aucacris_bullocki		XO		24	23	12
Orthoptera	Acrididae	Baeacris	Baeacris_punctulatus	gonochorous	XY		22	22	11
Orthoptera	Acrididae	Belosacris	Belosacris_coccineipes		XO		24	23	12
Orthoptera	Acrididae	Bucephalacris	Bucephalacris_bohlsii		XO		22	21	11
Orthoptera	Ommexechidae	Calcitrena	Calcitrena_maculosa		XO		24	23	12
Orthoptera	Acrididae	Carbonellacris	Carbonellacris_grossa		XO		24	23	12
Orthoptera	Romaleidae	Chariacris	Chariacris_miniacea		XO		24	23	12
Orthoptera	Acrididae	Chirista	Chirista_compta	gonochorous	XO		24	23	12
Orthoptera	Acrididae	Chlorus	Chlorus_bolivianus		XO		20	19	10
Orthoptera	Acrididae	Chlorus	Chlorus_borrelli		XO		22	21	11
Orthoptera	Acrididae	Chlorus	Chlorus_sp._1		XO		20	19	10

Orthoptera	Acrididae	Chlorus	Chlorus_vittatus		XO		24	23	12
Orthoptera	Romaleidae	Chromacris	Chromacris_miles		XO		24	23	12
Orthoptera	Romaleidae	Chromacris	Chromacris_peruviana		XO		24	23	12
Orthoptera	Romaleidae	Chromacris	Chromacris_speciosa		XO		24	23	12
Orthoptera	Ommexechidae	Clarazella	Clarazella_bimaculata		XO		24	23	12
Orthoptera	Ommexechidae	Clarazella	Clarazella_patagona		XO		24	23	12
Orthoptera	Acrididae	Cocytolettix	Cocytolettix_argentina		XO		24	23	12
Orthoptera	Acrididae	Cocytolettix	Cocytolettix_intermedia		XO		24	23	12
Orthoptera	Acrididae	Cocytolettix	Cocytolettix_pulchripennis		XO		24	23	12
Orthoptera	Acrididae	Cocytolettix	Cocytolettix_sp._1		XO		24	23	12
Orthoptera	Ommexechidae	Conometopus	Conometopus_sulcaticollis		XO		26	25	13
Orthoptera	Acrididae	Cornops	Cornops_aquaticum		XO		24	23	12
Orthoptera	Acrididae	Cornops	Cornops_frenatum		XO		24	23	12
Orthoptera	Romaleidae	Coryacris	Coryacris_angustipennis		XO		24	23	12
Orthoptera	Acrididae	Coryphosima	Coryphosima_stenoptera	gonochorous	XO		24	23	12
Orthoptera	Acrididae	Covasacris	Covasacris_sp.		XO		24	23	12
Orthoptera	Ommexechidae	Cumainocloidus	Cumainocloidus_cordillerae		XO		24	23	12
Orthoptera	Ommexechidae	Descampsacris	Descampsacris_serrulata		XO		24	23	12
Orthoptera	Acrididae	Dichromatos	Dichromatos_corupa	gonochorous	XY	X1X1X2X2/X1X2Y	22	21	11
Orthoptera	Acrididae	Dichromatos	Dichromatos_lilloanus	gonochorous	XY	X1X1X2X2/X1X2Y	22	21	11
Orthoptera	Acrididae	Dichromatos	Dichromatos_montanus	gonochorous	XY	X1X1X2X2/X1X2Y	22	21	11
Orthoptera	Acrididae	Dichromatos	Dichromatos_schrottkyi	gonochorous	XY	X1X1X2X2/X1X2Y	22	21	11
Orthoptera	Acrididae	Dichromorpha	Dichromorpha_australis		XO		24	23	12
Orthoptera	Acrididae	Dichroplus	Dichroplus_alejomesai		XO		24	23	12
Orthoptera	Acrididae	Dichroplus	Dichroplus_auriventris		XO		24	23	12
Orthoptera	Acrididae	Dichroplus	Dichroplus_bergi		XY		22	22	11
Orthoptera	Acrididae	Dichroplus	Dichroplus_conspersus		XO		24	23	12
Orthoptera	Acrididae	Dichroplus	Dichroplus_democraticus		XO		24	23	12
Orthoptera	Acrididae	Dichroplus	Dichroplus_dubius		complex XY	X1X1X2X2/X1X2Y	22	21	11
Orthoptera	Acrididae	Dichroplus	Dichroplus_elongatus		XO		24	23	12
Orthoptera	Acrididae	Dichroplus	Dichroplus_exilis		XO		24	23	12

Orthoptera	Acrididae	Dichroplus	Dichroplus_fuscus	XO		24	23	12
Orthoptera	Acrididae	Dichroplus	Dichroplus_fuscus.2	XO		20	19	10
Orthoptera	Acrididae	Dichroplus	Dichroplus_maculipennis	XO		24	23	12
Orthoptera	Acrididae	Dichroplus	Dichroplus_mantiqueirae	XO		24	23	12
Orthoptera	Acrididae	Dichroplus	Dichroplus_misionensis	XO		24	23	12
Orthoptera	Acrididae	Dichroplus	Dichroplus_obsurus	XY		18	18	9
Orthoptera	Acrididae	Dichroplus	Dichroplus_paraelongatus	XO		24	23	12
Orthoptera	Acrididae	Dichroplus	Dichroplus_paraguayensis	XY		20	20	10
Orthoptera	Acrididae	Dichroplus	Dichroplus_patruelis	XO		22	21	11
Orthoptera	Acrididae	Dichroplus	Dichroplus_piceomaculatus	XY		22	22	11
Orthoptera	Acrididae	Dichroplus	Dichroplus_porteri	XY		22	22	11
Orthoptera	Acrididae	Dichroplus	Dichroplus_pratensis	XO		20	19	10
Orthoptera	Acrididae	Dichroplus	Dichroplus_pseudopunctulatus	XO		24	23	12
Orthoptera	Acrididae	Dichroplus	Dichroplus_punctulatus	XO		24	23	12
Orthoptera	Acrididae	Dichroplus	Dichroplus_robustulus	XO		24	23	12
Orthoptera	Acrididae	Dichroplus	Dichroplus_robustus	complex XY	X1X1X2X2/X1X2Y	22	21	11
Orthoptera	Acrididae	Dichroplus	Dichroplus_schulzi	XO		24	23	12
Orthoptera	Acrididae	Dichroplus	Dichroplus_silveiraguidoi	XY		8	8	4
Orthoptera	Acrididae	Dichroplus	Dichroplus_sp._11	XO		24	23	12
Orthoptera	Acrididae	Dichroplus	Dichroplus_sp._12	XY		20	20	10
Orthoptera	Acrididae	Dichroplus	Dichroplus_sp._13	XY		20	20	10
Orthoptera	Acrididae	Dichroplus	Dichroplus_sp._14	XY		22	22	11
Orthoptera	Acrididae	Dichroplus	Dichroplus_sp._15	XY		20	20	10
Orthoptera	Acrididae	Dichroplus	Dichroplus_sp._16	XY		22	22	11
Orthoptera	Acrididae	Dichroplus	Dichroplus_vittatus	XY		20	20	10
Orthoptera	Acrididae	Dichroplus	Dichroplus_vittatus.2	XY		18	18	9
Orthoptera	Acrididae	Dichroplus	Dichroplus_vittigerum	XY		18	18	9
Orthoptera	Romaleidae	Diponthus	Diponthus_clarazianus	XO		24	23	12
Orthoptera	Romaleidae	Diponthus	Diponthus_communis	XY		22	22	11
Orthoptera	Romaleidae	Diponthus	Diponthus_dispar	XO		22	21	11
Orthoptera	Romaleidae	Diponthus	Diponthus_electus	XO		22	21	11

Orthoptera	Romaleidae	Diponthus	Diponthus_maculiferus		XO		22	21	11
Orthoptera	Romaleidae	Diponthus	Diponthus_prope_communis		XO		24	23	12
Orthoptera	Romaleidae	Diponthus	Diponthus_sp.		XO		24	23	12
Orthoptera	Romaleidae	Elaeochlora	Elaeochlora_basalis		XO		24	23	12
Orthoptera	Romaleidae	Elaeochlora	Elaeochlora_brachyptera		XO		24	23	12
Orthoptera	Romaleidae	Elaeochlora	Elaeochlora_sp.		XO		24	23	12
Orthoptera	Romaleidae	Elaeochlora	Elaeochlora_trilineata		XO		24	23	12
Orthoptera	Romaleidae	Elaeochlora	Elaeochlora_viridicata		XO		24	23	12
Orthoptera	Tristiridae	Elysiacris	Elysiacris_angusticollis		XO		22	21	11
Orthoptera	Phalangopsidae	Endecous	Endecous_onthophagus	gonochorous	XO		20	19	10
Orthoptera	Phalangopsidae	Endecous	Endecous_itatibensis	gonochorous	XO		20	19	10
Orthoptera	Phalangopsidae	Endecous	Endecous_cavernicolus	gonochorous	XO		22	21	11
Orthoptera	Phalangopsidae	Endecous	Endecous_batariensis	gonochorous	XO		22	21	11
Orthoptera	Phalangopsidae	Endecous	Endecous_alejomesai	gonochorous	XO		22	21	11
Orthoptera	Acrididae	Eucephalacris	Eucephalacris_borellii		XO		24	23	12
Orthoptera	Acrididae	Eujivarus	Eujivarus_fusiformis		XO		22	21	11
Orthoptera	Acrididae	Eujivarus	Eujivarus_sp._A		XO		22	21	11
Orthoptera	Acrididae	Eujivarus	Eujivarus_sp._B		XO		22	21	11
Orthoptera	Acrididae	Eujivarus	Eujivarus_sp._C		XO		22	21	11
Orthoptera	Acrididae	Eujivarus	Eujivarus_vittatus		XO		24	23	12
Orthoptera	Acrididae	Eulampiacris	Eulampiacris_leucoptera		XO		24	23	12
Orthoptera	Acrididae	Euplectrotettix	Euplectrotettix_sp._1		XO		24	23	12
Orthoptera	Acrididae	Euplectrotettix	Euplectrotettix_sp._2		XO		24	23	12
Orthoptera	Acrididae	Euplectrotettix	Euplectrotettix_sp._3		XO		24	23	12
Orthoptera	Acrididae	Eurotettix	Eurotettix_lilloanus		complex XY	X1X1X2X2/X1X2Y	22	21	11
Orthoptera	Acrididae	Eurotettix	Eurotettix_minor		XY		22	22	11
Orthoptera	Acrididae	Eurotettix	Eurotettix_schrottkyi		complex XY	X1X1X2X2/X1X2Y	22	21	11
Orthoptera	Acrididae	Eurotettix	Eurotettix_sp._1		complex XY	X1X1X2X2/X1X2Y	22	21	11
Orthoptera	Acrididae	Eurotettix	Eurotettix_sp._2		complex XY	X1X1X2X2/X1X2Y	22	21	11
Orthoptera	Acrididae	Eusitalces	Eusitalces_sp._A		XO		24	23	12
Orthoptera	Acrididae	Eusitalces	Eusitalces_vulneratus		XO		24	23	12

Orthoptera	Romaleidae	Eutropidacris	Eutropidacris_collares		XO	24	23	12
Orthoptera	Acrididae	Eutryxalis	Eutryxalis_sp.		XO	24	23	12
Orthoptera	Acrididae	Fenestra	Fenestra_bohlsii		XO	24	23	12
Orthoptera	Ommexechidae	Graea	Graea_horrida		XO	24	23	12
Orthoptera	Gryllotalpidae	Gryllotalpa	Gryllotalpa_marismortui		XO	24	23	12
Orthoptera	Gryllotalpidae	Gryllotalpa	Gryllotalpa_tali		XO	20	19	10
Orthoptera	Gryllotalpidae	Gryllotalpa	Gryllotalpa_gryllotalpa		XY	12	12	6
Orthoptera	Gryllotalpidae	Gryllotalpa	Gryllotalpa_stepposa		XY	12	12	6
Orthoptera	Gryllotalpidae	Gryllotalpa	Gryllotalpa_unispina		XO	20	19	10
Orthoptera	Acrididae	Haroldgrantia	Haroldgrantia_lignosa		XO	24	23	12
Orthoptera	Acrididae	Hyalopteryx	Hyalopteryx_rufipennis		XO	24	23	12
Orthoptera	Tristiridae	Illapelia	Illapelia_penai		XO	24	23	12
Orthoptera	Acrididae	Isonyx	Isonyx_paragauyensis		XO	24	23	12
Orthoptera	Acrididae	Isonyx	Isonyx_sp._1		XO	24	23	12
Orthoptera	Tettigoniidae	Isophya	Isophya_modestior	gonochorous	XO	32	31	16
Orthoptera	Tettigoniidae	Isophya	Isophya_rammei	gonochorous	XO	32	31	16
Orthoptera	Tettigoniidae	Isophya	Isophya_rectipennis	gonochorous	XO	32	31	16
Orthoptera	Tettigoniidae	Isophya	Isophya_kalishevskii	gonochorous	XO	32	31	16
Orthoptera	Tettigoniidae	Isophya	Isophya_schneideri	gonochorous	XO	32	31	16
Orthoptera	Tettigoniidae	Isophya	Isophya_speciosa	gonochorous	XO	32	31	16
Orthoptera	Tettigoniidae	Isophya	Isophya_hospodat	gonochorous	XO	32	31	16
Orthoptera	Tettigoniidae	Isophya	Isophya_tosevski	gonochorous	XO	32	31	16
Orthoptera	Tettigoniidae	Isophya	Isophya_andreevae	gonochorous	XO	32	31	16
Orthoptera	Tettigoniidae	Isophya	Isophya_miksici	gonochorous	XO	32	31	16
Orthoptera	Tettigoniidae	Isophya	Isophya_plevnensis	gonochorous	XO	32	31	16
Orthoptera	Tettigoniidae	Isophya	Isophya_pravdini pravdini	gonochorous	XO	32	31	16
Orthoptera	Tettigoniidae	Isophya	Isophya_pravdini adamovici	gonochorous	XO	32	31	16
Orthoptera	Tettigoniidae	Isophya	Isophya_modesta longicaudata	gonochorous	XO	32	31	16
Orthoptera	Tettigoniidae	Isophya	Isophya_rhodopensis	gonochorous	XO	32	31	16
Orthoptera	Tettigoniidae	Isophya	Isophya_petkovi	gonochorous	XO	32	31	16
Orthoptera	Tettigoniidae	Isophya	Isophya_kisi	gonochorous	XO	32	31	16

Orthoptera	Tettigoniidae	Isophya	Isophya_bureschi	gonochorous	XO		32	31	16
Orthoptera	Tettigoniidae	Isophya	Isophya_gulae	gonochorous	XO		32	31	16
Orthoptera	Tettigoniidae	Isophya	Isophya_obtusa	gonochorous	XO		32	31	16
Orthoptera	Tettigoniidae	Isophya	Isophya_altaica	gonochorous	XO		32	31	16
Orthoptera	Tettigoniidae	Isophya	Isophya_camptoxypha	gonochorous	XO		32	31	16
Orthoptera	Tettigoniidae	Isophya	Isophya_krausii	gonochorous	XO		32	31	16
Orthoptera	Tettigoniidae	Isophya	Isophya_pienensis	gonochorous	XO		32	31	16
Orthoptera	Tettigoniidae	Isophya	Isophya_hemiptera	gonochorous	XY	neo-XY	30	30	15
Orthoptera	Acrididae	Jodacris	Jodacris_chapadensis		XO		20	19	10
Orthoptera	Acrididae	Jodacris	Jodacris_ferrugineus		XO		20	19	10
Orthoptera	Acrididae	Jodacris	Jodacris_furcillata		XO		20	19	10
Orthoptera	Lentulidae	Karruacris	Karruacris_browni	gonochorous	XY	X1X1X2X2/X1X2Y	NA	NA	
Orthoptera	Acrididae	Lamiacris	Lamiacris_migroguttata		XO		24	23	12
Orthoptera	Acrididae	Laplatacris	Laplatacris_dispar		XO		24	23	12
Orthoptera	Acrididae	Laplatacris	Laplatacris_sp._1		XO		24	23	12
Orthoptera	Acrididae	Leiotettix	Leiotettix_politus	gonochorous	XY	X1X1X2X2/X1X2Y	14	NA	7
Orthoptera	Acrididae	Leiotettix	Leiotettix_flavipes		XY		22	22	11
Orthoptera	Acrididae	Leiotettix	Leiotettix_politus		XY		14	14	7
Orthoptera	Acrididae	Leiotettix	Leiotettix_politus.2		complex XY	X1X1X2X2/X1X2Y	14	13	7
Orthoptera	Acrididae	Leiotettix	Leiotettix_pulcher		XY		22	22	11
Orthoptera	Acrididae	Leiotettix	Leiotettix_sanguineus		XO		24	23	12
Orthoptera	Acrididae	Leiotettix	Leiotettix_sp._1		complex XY	X1X1X2X2/X1X2Y	16	15	8
Orthoptera	Acrididae	Leiotettix	Leiotettix_sp._2		XY		18	18	9
Orthoptera	Acrididae	Leiotettix	Leiotettix_sp._3		XO		24	23	12
Orthoptera	Acrididae	Leiotettix	Leiotettix_viridis		XO		24	23	12
Orthoptera	Acrididae	Leptysma	Leptysma_dorsalis		XO		24	23	12
Orthoptera	Acrididae	Leptysmina	Leptysmina_pallida		XO		24	23	12
Orthoptera	Acrididae	Leptysmina	Leptysmina_sp._1		XO		24	23	12
Orthoptera	Acrididae	Machaeropeles	Machaeropeles_rostratum		XO		24	23	12
Orthoptera	Pauliniidae	Marellia	Marellia_remipes		XO		24	23	12
Orthoptera	Acrididae	Mastusia	Mastusia_quadricarinata		XO		24	23	12

Orthoptera	Acrididae	Meloscirtus	Meloscirtus_montanus	XO	24	23	12
Orthoptera	Acrididae	Meloscirtus	Meloscirtus_sp._1	XO	24	23	12
Orthoptera	Acrididae	Metaleptea	Metaleptea_brevicornis_adspersa	XO	24	23	12
Orthoptera	Acrididae	Nahuelia	Nahuelia_rubriventris	XO	24	23	12
Orthoptera	Acrididae	Neopedies	Neopedies_brunneri	XO	24	23	12
Orthoptera	Acrididae	Neopedies	Neopedies_sp._1	XO	24	23	12
Orthoptera	Acrididae	Neopedies	Neopedies_sp._2	XO	24	23	12
Orthoptera	Acrididae	Neopedies	Neopedies_sp._3	XO	24	23	12
Orthoptera	Acrididae	Neopedies	Neopedies_sp._4	XO	24	23	12
Orthoptera	Ommexechidae	Neuquina	Neuquina_fictor	XY	22	22	11
Orthoptera	Acrididae	Notopomala	Notopomala_glaucipes	XO	24	23	12
Orthoptera	Acrididae	Omalotettix	Omalotettix_obliquum	XO	22	21	11
Orthoptera	Acrididae	Ommatolampis	Ommatolampis_perspicillata	XO	24	23	12
Orthoptera	Ommexechidae	Ommexecha	Ommexecha_sp.	XO	24	23	12
Orthoptera	Ommexechidae	Ommexecha	Ommexecha_virens	XO	24	23	12
Orthoptera	Ommexechidae	Ommexechia	Ommexechia_germari	XO	22	21	11
Orthoptera	Pyrgomorphidae	Omura	Omura_congrua	XO	20	19	10
Orthoptera	Acrididae	Orphula	Orphula_sp.	XO	24	23	12
Orthoptera	Acrididae	Orphulella	Orphulella_concinnula	XO	24	23	12
Orthoptera	Acrididae	Orphulella	Orphulella_punctata	XO	24	23	12
Orthoptera	Acrididae	Orphulella	Orphulella_sp.	XO	24	23	12
Orthoptera	Acrididae	Orphulina	Orphulina_pulchella	XO	24	23	12
Orthoptera	Acrididae	Osmilia	Osmilia_flavolineata	XO	24	23	12
Orthoptera	Acrididae	Oxyblepta	Oxyblepta_sp.	XO	24	23	12
Orthoptera	Acrididae	Oxybleptella	Oxybleptella_sagitta	XO	24	23	12
Orthoptera	Ommexechidae	Pachyosa	Pachyosa_signata	XY	22	22	11
Orthoptera	Acrididae	Paraorphula	Paraorphula_graminea	XO	24	23	12
Orthoptera	Acrididae	Parapellopedon	Parapellopedon_instabilis	XO	24	23	12
Orthoptera	Acrididae	Parapellopedon	Parapellopedon_sp.	XO	24	23	12
Orthoptera	Acrididae	Parascopas	Parascopas_exertus	XO	22	21	11
Orthoptera	Acrididae	Parascopas	Parascopas_obesus	XO	24	23	12

Orthoptera	Acrididae	Parascopas	Parascopas_sanguineus		XO		24	23	12
Orthoptera	Acrididae	Parascopas	Parascopas_similis		XO		24	23	12
Orthoptera	Acrididae	Paratylotropidia	Paratylotropidia_morsei	gonochorous	XY	X1X1X2X2/X1X2Y	NA	NA	
Orthoptera	Acrididae	Paropaon	Paropaon_laevifrons		XO		24	23	12
Orthoptera	Acrididae	Paropaon	Paropaon_pilosus_tingomariae		XO		24	23	12
Orthoptera	Pauliniidae	Paulinia	Paulinia_acuminata		XO		24	23	12
Orthoptera	Acrididae	Pedies	Pedies_andeanus		XO		22	21	11
Orthoptera	Acrididae	Pedies	Pedies_sp._1		XO		22	21	11
Orthoptera	Acrididae	Pedies	Pedies_sp._2		XO		22	21	11
Orthoptera	Acrididae	Pedies	Pedies_sp._3		XO		22	21	11
Orthoptera	Acrididae	Pedies	Pedies_sp._4		XO		24	23	12
Orthoptera	Tristiridae	Peplacris	Peplacris_recutita		XO		22	21	11
Orthoptera	Romaleidae	Prionolopha	Prionolopha_serrata		XO		24	23	12
Orthoptera	Romaleidae	Procolpia	Procolpia_minor		XO		24	23	12
Orthoptera	Acrididae	Propedies	Propedies_bilobus		XO		24	23	12
Orthoptera	Acrididae	Propedies	Propedies_bipunctatus		XO		24	23	12
Orthoptera	Acrididae	Propedies	Propedies_fusiformis		XO		24	23	12
Orthoptera	Acrididae	Propedies	Propedies_olivaceus		XO		24	23	12
Orthoptera	Acrididae	Propedies	Propedies_sanguineus		XO		24	23	12
Orthoptera	Acrididae	Propedies	Propedies_sp._1		XO		24	23	12
Orthoptera	Acrididae	Pseudoscopas	Pseudoscopas_nigrigena		XO		24	23	12
Orthoptera	Acrididae	Pseudoscopas	Pseudoscopas_sp._1		XO		24	23	12
Orthoptera	Acrididae	Pseudoscopas	Pseudoscopas_sp._2		XO		24	23	12
Orthoptera	Acrididae	Pseudoscopas	Pseudoscopas_sp._3		XO		24	23	12
Orthoptera	Acrididae	Pseudoscopas	Pseudoscopas_sp._4		XO		24	23	12
Orthoptera	Acrididae	Pseudoscopas	Pseudoscopas_sp._5		XO		24	23	12
Orthoptera	Acrididae	Pseudoscopas	Pseudoscopas_sp._6		XO		24	23	12
Orthoptera	Acrididae	Pseudoscopas	Pseudoscopas_sp._7		XO		24	23	12
Orthoptera	Acrididae	Pseudoscopas	Pseudoscopas_sp._8		XO		24	23	12
Orthoptera	Acrididae	Psiloscirtus	Psiloscirtus_bolivianus		XO		24	23	12
Orthoptera	Acrididae	Psiloscirtus	Psiloscirtus_olivaceus		XO		24	23	12

Orthoptera	Acrididae	Psiloscirtus	Psiloscirtus_sp._A		XO		24	23	12
Orthoptera	Acrididae	Pycnosarcus	Pycnosarcus_atavus		XO		18	17	9
Orthoptera	Acrididae	Ronderosia	Ronderosia_dubius	gonochorous	XY	X1X1X2X2/X1X2Y	22	21	11
Orthoptera	Acrididae	Ronderosia	Ronderosia_robustus	gonochorous	XY	X1X1X2X2/X1X2Y	22	21	11
Orthoptera	Acrididae	Schistocerca	Schistocerca_cancellata		XO		24	23	12
Orthoptera	Acrididae	Schistocerca	Schistocerca_flavofasciata		XO		24	23	12
Orthoptera	Acrididae	Schistocerca	Schistocerca_pallens		XO		24	23	12
Orthoptera	Acrididae	Schistocerca	Schistocerca_paraensis		XO		24	23	12
Orthoptera	Acrididae	Schistocerca	Schistocerca_sp.		XO		24	23	12
Orthoptera	Acrididae	Scotussa	Scotussa_daguerrei	gonochorous	XY	X1X1X2X2/X1X2Y	22	21	11
Orthoptera	Acrididae	Scotussa	Scotussa_cliens		XO		22	21	11
Orthoptera	Acrididae	Scotussa	Scotussa_daguerrei		complex XY	X1X1X2X2/X1X2Y	22	21	11
Orthoptera	Acrididae	Scotussa	Scotussa_delicatula		XY		16	16	8
Orthoptera	Acrididae	Scotussa	Scotussa_impudica		XO		24	23	12
Orthoptera	Acrididae	Scotussa	Scotussa_lemniscata		XO		24	23	12
Orthoptera	Acrididae	Scotussa	Scotussa_liebermanni		XO		22	21	11
Orthoptera	Acrididae	Scotussa	Scotussa_sp._1		XO		24	23	12
Orthoptera	Acrididae	Scyllina	Scyllina_humilis		XO		24	23	12
Orthoptera	Acrididae	Scyllina	Scyllina_signatipennis		XY		22	22	11
Orthoptera	Acrididae	Scyllina	Scyllina_sp.		XO		24	23	12
Orthoptera	Acrididae	Scyllinops	Scyllinops_brunneri		XO		24	23	12
Orthoptera	Acrididae	Scyllinops	Scyllinops_pallida		XO		24	23	12
Orthoptera	Acrididae	Scyllinops	Scyllinops_sp._1		XO		24	23	12
Orthoptera	Acrididae	Scyllinops	Scyllinops_sp._2		XO		24	23	12
Orthoptera	Romaleidae	Securigera	Securigera_acutangula		XO		24	23	12
Orthoptera	Acrididae	Silvitettix	Silvitettix_concolor		XO		24	23	12
Orthoptera	Acrididae	Sinipta	Sinipta_acuta		XO		24	23	12
Orthoptera	Acrididae	Sinipta	Sinipta_dalmani		XO		24	23	12
Orthoptera	Acrididae	Sinipta	Sinipta_maldonadoi		XO		24	23	12
Orthoptera	Acrididae	Sitalces	Sitalces_dorsalis		XO		24	23	12
Orthoptera	Acrididae	Sitalces	Sitalces_infuscatus		XO		24	23	12

Orthoptera	Acrididae	Sitalces	Sitalces_volxemi	XO	20	19	10
Orthoptera	Ommexechidae	Spathalium	Spathalium_audouini	XY	22	22	11
Orthoptera	Acrididae	Staurorhectus	Staurorhectus_longicornis	XO	24	23	12
Orthoptera	Acrididae	Stenopola	Stenopola_bohlsii	XO	24	23	12
Orthoptera	Acrididae	Stenopola	Stenopola_boliviana	XO	24	23	12
Orthoptera	Acrididae	Stenopola	Stenopola_dorsalis	XO	24	23	12
Orthoptera	Acrididae	Stenopola	Stenopola_pallida	XO	22	21	11
Orthoptera	Acrididae	Stenopola	Stenopola_rubrifons	XO	24	23	12
Orthoptera	Acrididae	Stereotettix	Stereotettix_sp._1	XO	24	23	12
Orthoptera	Acrididae	Tetrataenia	Tetrataenia_surinama	XO	20	19	10
Orthoptera	Ommexechidae	Tetrixocephalus	Tetrixocephalus_chilensis	XO	24	23	12
Orthoptera	Ommexechidae	Tetrixocephalus	Tetrixocephalus_micropterus	XO	24	23	12
Orthoptera	Ommexechidae	Tetrixocephalus	Tetrixocephalus_sergioi	XO	24	23	12
Orthoptera	Ommexechidae	Tetrixocephalus	Tetrixocephalus_sp.	XO	24	23	12
Orthoptera	Ommexechidae	Tetrixocephalus	Tetrixocephalus_willemsei	XY	22	22	11
Orthoptera	Acrididae	Trimerotropis	Trimerotropis_ochraceipennis	XO	24	23	12
Orthoptera	Acrididae	Trimerotropis	Trimerotropis_pallidipennis	XO	24	23	12
Orthoptera	Tristiridae	Tropidostethus	Tropidostethus_bicarinatus	XO	22	21	11
Orthoptera	Romaleidae	Xestotrachelus	Xestotrachelus_robustus	XO	24	23	12
Orthoptera	Acrididae	Xiphiola	Xiphiola_borellii	XO	24	23	12
Orthoptera	Romaleidae	Xyleus	Xyleus_attenuatus	XO	24	23	12
Orthoptera	Romaleidae	Xyleus	Xyleus_discoideus	XO	24	23	12
Orthoptera	Romaleidae	Xyleus	Xyleus_gracilis	XO	24	23	12
Orthoptera	Romaleidae	Xyleus	Xyleus_insignis	XO	24	23	12
Orthoptera	Romaleidae	Xyleus	Xyleus_laevipes	XY	22	22	11
Orthoptera	Romaleidae	Xyleus	Xyleus_modestus	XO	24	23	12
Orthoptera	Romaleidae	Xyleus	Xyleus_sp._1	XO	24	23	12
Orthoptera	Romaleidae	Xyleus	Xyleus_sp._2	XO	24	23	12
Orthoptera	Romaleidae	Xyleus	Xyleus_sp._3	XO	24	23	12
Orthoptera	Romaleidae	Zoniopoda	Zoniopoda_hempeli	XO	24	23	12
Orthoptera	Romaleidae	Zoniopoda	Zoniopoda_iheringi	XY	22	22	11

Orthoptera	Romaleidae	Zoniopoda	Zoniopoda_juncorum		XO	24	23	12
Orthoptera	Romaleidae	Zoniopoda	Zoniopoda_omnicolor		XO	24	23	12
Orthoptera	Romaleidae	Zoniopoda	Zoniopoda_similis		XO	24	23	12
Orthoptera	Romaleidae	Zoniopoda	Zoniopoda_tarsata		XO	24	23	12
Orthoptera	Acrididae	Zygoclistron	Zygoclistron_falconinum		XY	20	20	10
Orthoptera	Acrididae	Zygoclistron	Zygoclistron_nasicum		XY	20	20	10
Orthoptera	Acrididae	Zygoclistron	Zygoclistron_trachystictum		XY	20	20	10
Phasmatodea	Phylliidae	Acanthoderus	Acanthoderus_grandis		XO	44	43	22
Phasmatodea	Phylliidae	Acanthoderus	Acanthoderus_inermis		XO	44	43	22
Phasmatodea	Phasmatidae	Acanthoxyla	Acanthoxyla_inermis			36	NA	18
Phasmatodea	Phasmatidae	Acanthoxyla	Acanthoxyla_fasciata	parthenogenetic		NA	NA	
Phasmatodea	Phasmatidae	Acanthoxyla	Acanthoxyla_geisovii	parthenogenetic		38	NA	19
Phasmatodea	Phasmatidae	Acanthoxyla	Acanthoxyla_huttoni	parthenogenetic		NA	NA	
Phasmatodea	Phasmatidae	Acanthoxyla	Acanthoxyla_intermedia	parthenogenetic		38	NA	19
Phasmatodea	Phasmatidae	Acanthoxyla	Acanthoxyla_prasina	parthenogenetic		NA	NA	
Phasmatodea	Phasmatidae	Acanthoxyla	Acanthoxyla_speciosa	parthenogenetic		NA	NA	
Phasmatodea	Phasmatidae	Acanthoxyla	Acanthoxyla_suteri	parthenogenetic		NA	NA	
Phasmatodea	Phasmidae	Acrophylla	Acrophylla_titan		XO	36	35	18
Phasmatodea	Phasmidae	Aplopus	Aplopus_mayeri		XO	36	35	18
Phasmatodea	Phylliidae	Bacillus	Bacillus_atticus			34	NA	17
Phasmatodea	Phylliidae	Bacillus	Bacillus_grandii_benazzii		XO	34	33	17
Phasmatodea	Phylliidae	Bacillus	Bacillus_grandii_grandii		XO	34	33	17
Phasmatodea	Phylliidae	Bacillus	Bacillus_lynceorum	parthenogenetic		51	NA	26
Phasmatodea	Phylliidae	Bacillus	Bacillus_rossius-grandii_benazzii			35	NA	18
Phasmatodea	Phylliidae	Bacillus	Bacillus_rossius-grandii_grandii			35	NA	18
Phasmatodea	Phylliidae	Bacillus	Bacillus_whitei	parthenogenetic		35	NA	18
Phasmatodea	Phylliidae	Bacillus	Bacillus_libanicus		XY	NA	NA	
Phasmatodea	Phylliidae	Bacillus	Bacillus_rossius		XO	36	35	18
Phasmatodea	Phylliidae	Bacillus	Bacillus_rossius			NA	36	18
Phasmatodea	Phasmidae	Baculum	Baculum_artemis			72	72	36
Phasmatodea	Phasmidae	Bostra	Bostra_sp.		XO	36	35	18

Phasmatodea	Phasmidae	Carausius	Carausius_morosus	parthenogenetic		64	NA	32
Phasmatodea	Phasmidae	Carausius	Carausius_furcillatus			NA	66	33
Phasmatodea	Phasmidae	Carausius	Carausius_furcillatus			NA	87	44
Phasmatodea	Phasmidae	Carausius	Carausius_juvenilis		XO	42	41	21
Phasmatodea	Phasmidae	Carausius	Carausius_morosus			NA	65	33
Phasmatodea	Phasmidae	Carausius	Carausius_rotundato-lobatus		XO	22	21	11
Phasmatodea	Phasmidae	Carausius	Carausius_sp.		XO	42	41	21
Phasmatodea	Phasmidae	Carausius	Carausius_theiseni			NA	41	21
Phasmatodea	Phasmidae	Carnacia	Carnacia_telesphorus		XO	44	43	22
Phasmatodea	Phasmatidae	Clitarchus	Clitarchus_hookeri			36	NA	18
Phasmatodea	Phasmidae	Clitumnus	Clitumnus_extradentatus		XO	38	37	19
Phasmatodea	Phasmidae	Clonistria	Clonistria_exornata		XO	34	33	17
Phasmatodea	Phylliidae	Clonopsis	Clonopsis_sp._1		XO	36	35	18
Phasmatodea	Phylliidae	Clonopsis	Clonopsis_sp._2			72	NA	36
Phasmatodea	Phylliidae	Clonopsis	Clonopsis_androgenes			44	NA	22
Phasmatodea	Phylliidae	Clonopsis	Clonopsis_felicitatis		XO	36	35	18
Phasmatodea	Phylliidae	Clonopsis	Clonopsis_gallica	parthenogenetic		54	NA	27
Phasmatodea	Phylliidae	Clonopsis	Clonopsis_maroccana		XO	22	21	11
Phasmatodea	Phylliidae	Clonopsis	Clonopsis_soumiae	parthenogenetic		72	NA	36
Phasmatodea	Phylliidae	Clonopsis	Clonopsis_gallica			56	56	28
Phasmatodea	Phasmidae	Ctenomorpha	Ctenomorpha_chronus		XY	30	30	15
Phasmatodea	Phasmidae	Ctenomorpha	Ctenomorpha_chronus		XY	32	32	16
Phasmatodea	Phasmidae	Ctenomorphodes	Ctenomorphodes_sp.2			40	NA	20
Phasmatodea	Phasmidae	Ctenomorphodes	Ctenomorphodes_tessulatus		XO	38	37	19
Phasmatodea	Phasmidae	Didymuria	Didymuria_violescens		XY	27	26	14
Phasmatodea	Phasmidae	Didymuria	Didymuria_violescens		XY	29	28	15
Phasmatodea	Phasmidae	Didymuria	Didymuria_violescens		XY	29	28	15
Phasmatodea	Phasmidae	Didymuria	Didymuria_violescens		XY	31	30	16
Phasmatodea	Phasmidae	Didymuria	Didymuria_violescens		XO	32	31	16
Phasmatodea	Phasmidae	Didymuria	Didymuria_violescens		XY	33	32	17
Phasmatodea	Phasmidae	Didymuria	Didymuria_violescens		XO|XY	33	33	17

Phasmatodea	Phasmidae	Didymuria	Didymuria_violescens		XO	36	35	18
Phasmatodea	Phasmidae	Didymuria	Didymuria_violescens		XO	38	37	19
Phasmatodea	Phasmidae	Didymuria	Didymuria_violescens		XO	40	39	20
Phasmatodea	Phasmidae	Didymuria	Didymuria_violescens		XO	40	39	20
Phasmatodea	Phasmidae	Dubreuilia	Dubreuilia_lineata		XO	26	25	13
Phasmatodea	Phasmidae	Dyme	Dyme_krugiana		XO	50	49	25
Phasmatodea	Phylliidae	Epibacillus	Epibacillus_lobipes			NA	36	18
Phasmatodea	Phasmidae	Extatosoma	Extatosoma_tiaratum		XO	36	35	18
Phasmatodea	Phasmidae	Extatosoma	Extatosoma_tiaratum		XO	38	37	19
Phasmatodea	Phasmidae	Genus7	Genus7_longiceps			42	NA	21
Phasmatodea	Phasmidae	Genus8	Genus8_sp1		XO	46	45	23
Phasmatodea	Phylliidae	Isagoras	Isagoras_schraderi		XY	34	34	17
Phasmatodea	Phylliidae	Isagoras	Isagoras_sp.		XO	48	47	24
Phasmatodea	Phylliidae	Isagoras	Isagoras_subaquilus		XO	28	27	14
Phasmatodea	Phasmidae	Lampionus	Lampionus_sp.?		XO	24	23	12
Phasmatodea	Phasmidae	Lampionus	Lampionus_sp.I		XO	30	29	15
Phasmatodea	Phasmidae	Lampionus	Lampionus_sp.III		XO	36	35	18
Phasmatodea	Phasmidae	Lampionus	Lampionus_sp.V		XO	36	35	18
Phasmatodea	Phylliidae	Leptynia	Leptynia_attenuata		XY	36	36	18
Phasmatodea	Phylliidae	Leptynia	Leptynia_caprai		XO	40	39	20
Phasmatodea	Phylliidae	Leptynia	Leptynia_montana		XO	38	37	19
Phasmatodea	Phylliidae	Leptynia	Leptynia_sp.		XO	40	39	20
Phasmatodea	Phylliidae	Leptynia	Leptynia_attenuata		XO	36	35	18
Phasmatodea	Phasmatidae	Medauroidea	Medauroidea_extradentata	parthenogenetic		38	NA	19
Phasmatodea	Phasmatidae	Medauroidea	Medauroidea_extradentata	gonochorous	XO	NA	37	19
Phasmatodea	Phasmidae	Menexenus	Menexenus_semiarmatus		XO	46	45	23
Phasmatodea	Phasmidae	Oncotophasma	Oncotophasma_sp.		XO	42	41	21
Phasmatodea	Phasmidae	Orxines	Orxines_macklotti		XO	38	37	19
Phasmatodea	Phasmidae	Pachymorpha	Pachymorpha_simplicipes			34	NA	17
Phasmatodea	Phasmidae	Pachymorpha	Pachymorpha_sp.3		XO	30	29	15
Phasmatodea	Phasmidae	Pachymorpha	Pachymorpha_squalida		XO	34	33	17

Phasmatodea	Phasmidae	Parasipyloidea	Parasipyloidea_annulatus		XO	30	29	15
Phasmatodea	Phasmidae	Parasipyloidea	Parasipyloidea_cercata		XO	40	39	20
Phasmatodea	Phasmidae	Parasipyloidea	Parasipyloidea_granulosa		XO	36	35	18
Phasmatodea	Phasmidae	Parasipyloidea	Parasipyloidea_sp2		XO	40	39	20
Phasmatodea	Phasmidae	Parasipyloidea	Parasipyloidea_sp3		XO	28	27	14
Phasmatodea	Phasmidae	Parasipyloidea	Parasipyloidea_sp4		XO	39	38	20
Phasmatodea	Phasmidae	Parasipyloidea	Parasipyloidea_sp5		XO	39	38	20
Phasmatodea	Phasmidae	Parasosibia	Parasosibia_parva		XO	54	53	27
Phasmatodea	Pseudophasmatidae	Peruphasma	Peruphasma_schultei	gonochorous	XO	44	43	22
Phasmatodea	Diapheromeridae	Phaenopharos	Phaenopharos_khaoyaiensis	parthenogenetic		70		35
Phasmatodea	Phylliidae	Phalces	Phalces_longiscaphus		XO	36	35	18
Phasmatodea	Phasmidae	Phibalosoma	Phibalosoma_phyllinum		XO	42	41	21
Phasmatodea	Phasmidae	Phobaeticus	Phobaeticus_sinetyi		XO	52	51	26
Phasmatodea	Phylliidae	Phyllium	Phyllium_bioculatum		XO	34	33	17
Phasmatodea	Phylliidae	Pijnackeria	Pijnackeria_hispanica		XO	38	37	19
Phasmatodea	Phylliidae	Pijnackeria	Pijnackeria_hispanica			57	NA	29
Phasmatodea	Phylliidae	Pijnackeria	Pijnackeria_hispanica			76	NA	38
Phasmatodea	Phylliidae	Pijnackeria	Pijnackeria_barbarae		XO	38	37	19
Phasmatodea	Phylliidae	Pijnackeria	Pijnackeria_lelongi		XO	38	37	19
Phasmatodea	Phylliidae	Pijnackeria	Pijnackeria_lucianae		XO	38	37	19
Phasmatodea	Phylliidae	Pijnackeria	Pijnackeria_masettii		XO	57	NA	29
Phasmatodea	Phylliidae	Pijnackeria	Pijnackeria_originis		XO	38	37	19
Phasmatodea	Phylliidae	Pijnackeria	Pijnackeria_hispanica			54	54	27
Phasmatodea	Phasmidae	Podacanthus	Podacanthus_typhon		XY	28	28	14
Phasmatodea	Phasmidae	Podacanthus	Podacanthus_viridiroseus		XO	36	35	18
Phasmatodea	Phasmidae	Podacanthus	Podacanthus_wilkinsoni		XO	36	35	18
Phasmatodea	Phylliidae	Prisopus	Prisopus_ariadne		XY	28	28	14
Phasmatodea	Phylliidae	Prisopus	Prisopus_berosus		XO	50	49	25
Phasmatodea	Phasmidae	Pseudobacteria	Pseudobacteria_sp.		XO	48	46	24
Phasmatodea	Phylliidae	Pseudophasma	Pseudophasma_menius		XO	24	23	12
Phasmatodea	Phasmidae	Sipyloidea	Sipyloidea_panaeticus		XO	22	21	11

Phasmatodea	Phasmidae	Sipyloidea	Sipyloidea_sipylus				80	80	40
Phasmatodea	Phasmidae	Sipyloidea	Sipyloidea_sp6				38	NA	19
Phasmatodea	Heteropterygidae	Sungaya	Sungaya_inexpectata	parthenogenetic			44	NA	22
Phasmatodea	Timematidae	Timema	Timema_poppensis	gonochorous			24	NA	12
Phasmatodea	Timematidae	Timema	Timema_shepardi	parthenogenetic			24	NA	12
Phasmatodea	Phasmidae	Tropiderus	Tropiderus_childrenii		XO		34	33	17
Phasmatodea	Phasmidae	Vetilia	Vetilia_enceladus		XY		38	38	19
Plecoptera	Perlidae	Calineuria	Calineuria_jezoensis		XO		26	25	13
Plecoptera	Perlodidae	Isogenus	Isogenus_alpinus				NA	24	12
Plecoptera	Perlodidae	Isogenus	Isogenus_fontium		complex XO	X1X1X2X2/X1X2OO	NA	26	13
Plecoptera	Perlodidae	Isogenus	Isogenus_imhoffi				NA	26	13
Plecoptera	Perlodidae	Isoperla	Isoperla_grammatica		complex XO	X1X1X2X2/X1X2OO	NA	26	13
Plecoptera	Perlodidae	Isoperla	Isoperla_rivulorum		complex XO	X1X1X2X2/X1X2OO	NA	26	13
Plecoptera	Perlidae	Perla	Perla_marginata		complex XO	X1X1X2X2/X1X2OO	24	22	12
Plecoptera	Perlidae	Perla	Perla_abdominalis		complex XO	X1X1X2X2/X1X2OO	NA	26	13
Plecoptera	Perlidae	Perla	Perla_baetica		complex XO	X1X1X2X2/X1X2OO	NA	26	13
Plecoptera	Perlidae	Perla	Perla_bipunctata		XO		22	21	11
Plecoptera	Perlidae	Perla	Perla_cephalotes		complex XO	X1X1X2X2/X1X2OO	NA	26	13
Plecoptera	Perlidae	Perla	Perla_immarginata		XY		10	10	5
Plecoptera	Perlidae	Perla	Perla_maxima		XO		20	19	10
Plecoptera	Perlodidae	Perlodes	Perlodes_intricala				NA	33	17
Plecoptera	Perlodidae	Perlodes	Perlodes_jurassica				NA	31	16
Plecoptera	Perlodidae	Perlodes	Perlodes_microcephala		complex XO		NA	27	14

Table S2 1C genome sizes for Polyneoptera. All new records were run on a Partec Cyflow SL_3 cytometer with *Periplaneta americana* (1C = 3338 Mbp) used as a standard. AGSD; Animal genome size database.

Order	Family	Genus	Species	Sex	GS (Mbp)	Rep	StDev	Source
Blattodea	Blattellidae	*Blattella*	*germanica*	F	2161.66	3	45.2	This manuscript
Blattodea	Blattellidae	*Blattella*	*germanica*	M	2090.7	3	32.5	This manuscript
Blattodea	Blattidae	*Periplaneta*	*fuliginosa*	-	4498.9	3	91.4	This manuscript
Mantodea	Mantidae	*Stegomantis*	*sp*	M	3461.51	1	-	This manuscript
Mantodea	Mantidae	*Thesprotia*	*graminis*	M	2071.5	1	-	This manuscript
Orthoptera	Acrididae	*Acantherus*	*piperatus*	F	14287.9	1	-	This manuscript
Orthoptera	Acrididae	*Acrolophitus*	*hirtipes*	F	14813.4	1	-	This manuscript
Orthoptera	Acrididae	*Arethea*	*sp*	M	8539.8	1	-	This manuscript
Orthoptera	Acrididae	*Arphia*	*simplex*	F	11823.3	2	744.25	This manuscript
Orthoptera	Acrididae	*Arphia*	*simplex*	M	11134.8	6	436.25	This manuscript
Orthoptera	Acrididae	*Bootettix*	*argentatus*	F	11998.2	2	25.79	This manuscript
Orthoptera	Acrididae	*Bootettix*	*argentatus*	M	11401.1	2	159.19	This manuscript
Orthoptera	Acrididae	*Brachystola*	*magna*	F	16807	1	-	This manuscript
Orthoptera	Acrididae	*Chortophaga*	*viridifasciata*	F	10435.6	4	409.95	This manuscript
Orthoptera	Acrididae	*Chortophaga*	*viridifasciata*	M	10279.7	3	168.65	This manuscript
Orthoptera	Acrididae	*Dactylotum*	*bicolor*	M	10211.2	1	-	This manuscript
Orthoptera	Acrididae	*Encoptolophus*	*costalis*	F	8449	1	-	This manuscript
Orthoptera	Acrididae	*Encoptolophus*	*costalis*	M	7971.5	3	96.42	This manuscript
Orthoptera	Acrididae	*Hadrotettix*	*trifasciatus*	F	18051.1	1	-	This manuscript
Orthoptera	Acrididae	*Hadrotettix*	*trifasciatus*	M	17495.7	2	1010.54	This manuscript
Orthoptera	Acrididae	*Hippuscus*	*ocelote*	F	13810.4	2	739.17	This manuscript
Orthoptera	Acrididae	*Hippuscus*	*ocelote*	M	11940.9	3	245.45	This manuscript
Orthoptera	Acrididae	*Lactista*	*azteca*	F	10007.5	2	12.84	This manuscript
Orthoptera	Acrididae	*Lactista*	*azteca*	M	9555.5	1	-	This manuscript
Orthoptera	Acrididae	*Leprus*	*wheeleri*	F	13968.1	2	58.8	This manuscript
Orthoptera	Acrididae	*Leprus*	*wheeleri*	M	13466.6	7	449.09	This manuscript
Orthoptera	Acrididae	*Melanoplus*	*bispinosus*	M	5813	1	-	This manuscript
Orthoptera	Acrididae	*Melanoplus*	*differentialis*	F	7096.2	3	56.75	This manuscript
Orthoptera	Acrididae	*Melanoplus*	*differentialis*	M	6668.9	3	61.97	This manuscript
Orthoptera	Acrididae	*Melanoplus*	*femurrubrum*	F	6011.2	6	403.34	This manuscript
Orthoptera	Acrididae	*Melanoplus*	*femurrubrum*	M	5277.1	3	88.29	This manuscript
Orthoptera	Acrididae	*Mermiria*	*bivittata*	F	15430.5	9	671.76	This manuscript
Orthoptera	Acrididae	*Mermiria*	*bivittata*	M	14496.9	5	213.22	This manuscript
Orthoptera	Acrididae	*Psinidia*	*amplicornis*	F	9325.4	4	209.71	This manuscript
Orthoptera	Acrididae	*Psinidia*	*amplicornis*	M	8824.1	2	334.41	This manuscript
Orthoptera	Acrididae	*Schistocerca*	*americana*	M	8259.15	1	-	This manuscript
Orthoptera	Acrididae	*Schistocerca*	*nitens*	F	9251.34	1	-	This manuscript
Orthoptera	Acrididae	*Schistocerca*	*obscura*	M	9318.86	1	-	This manuscript
Orthoptera	Acrididae	*Spharagemon*	*cristatum*	F	10129.4	2	24.02	This manuscript
Orthoptera	Acrididae	*Spharagemon*	*cristatum*	M	9774.37	1	-	This manuscript
Orthoptera	Acrididae	*Spharagemon*	*equale*	M	12260.4	1	-	This manuscript
Orthoptera	Acrididae	*Syrbula*	*admirabilis*	F	9589.9	1	-	This manuscript
Orthoptera	Acrididae	*Syrbula*	*admirabilis*	M	9201	1	-	This manuscript
Orthoptera	Acrididae	*Syrbula*	*montezuma*	F	11765.8	1	-	This manuscript
Orthoptera	Acrididae	*Syrbula*	*montezuma*	M	11151.9	2	494.83	This manuscript
Orthoptera	Acrididae	*Trachyrachys*	*kiowa*	F	9357.4	1	-	This manuscript
Orthoptera	Acrididae	*Trachyrachys*	*kiowa*	M	8842.2	3	68.59	This manuscript
Orthoptera	Acrididae	*Trimerotropis*	*pallidipennis*	F	9219	1	-	This manuscript
Orthoptera	Acrididae	*Trimerotropis*	*pallidipennis*	M	9112.3	2	197.87	This manuscript
Orthoptera	Acrididae	*Trimerotropis*	*sp*	F	12134.2	1	-	This manuscript
Orthoptera	Acrididae	*Xanthippus*	*corallipes*	F	13759.3	1	-	This manuscript
Orthoptera	Acrididae	*Xanthippus*	*corallipes*	M	12809.9	1	-	This manuscript
Orthoptera	Acrididae	*Xanthippus*	*corallipes pantherinus*	F	12765.2	1	-	This manuscript
Orthoptera	Tettigoniidae	*Neobarrettia*	*spinosa*	F	5328.1	2	49.96	This manuscript
Orthoptera	Tettigoniidae	*Neobarrettia*	*spinosa*	M	4755.3	1	-	This manuscript
Orthoptera	Tettigoniidae	*Pediodectes*	*sp*	-	9080.7	1	-	This manuscript
Orthoptera	Tettigoniidae	*Pediodectes*	*tinkhami?*	M	7795.83	1	-	This manuscript
Orthoptera	Tettigoniidae	*Scudderia*	*sp*	-	13079.3	2	79.25	This manuscript
Phamatodea	Diapheromeridae	*Megaphasma*	*dentricus*	M	2774.1	1	-	This manuscript
Phamatodea	Pseudophasmatidae	*Anisomorpha*	*buprestoides*	M	2944.1	2	40.43	This manuscript
Blattodea	Blaberidae	*Blaberus*	*fuscus*		3286.08			AGSD

Blattodea	Blaberidae	*Blaptica*	*dubia*		4440.12			AGSD
Blattodea	Blaberidae	*Nauphoeta*	*cinerea*		5036.7			AGSD
Blattodea	Blatellidae	*Blatella*	*nipponica*		2083.14			AGSD
Blattodea	Blattellidae	*Blattella*	*germanica*		1956			AGSD
Blattodea	Blattidae	*Blatta*	*orientalis*		2963.34			AGSD
Blattodea	Blattidae	*Periplaneta*	*americana*		2660.16			AGSD
Blattodea	Cryptocercidae	*Cryptocercus*	*kyebangensis*		1134.48			AGSD
Blattodea	Cryptocercidae	*Cryptocercus*	*punctulatus*		1290.96			AGSD
Blattodea	Blaberidae	*Panchlora*	*nivea*		1486.56			AGSD
Blattodea	Blattellidae	*Parcoblatta*	*pensylvanica*		1026.9			AGSD
Blattodea	Blattidae	*Periplaneta*	*americana*		3334.98			AGSD
Dermaptera	Labiduridae	*Labidura*	*riparia*		518.34			AGSD
Embiidina	Oligotomidae	*Oligotoma*	*saundersii*		2601.48			AGSD
Isoptera	Hodotermitidae	*Hodotermes*	*mossambicus*		978			AGSD
Isoptera	Kalotermitidae	*Glyptotermes*	*fuscus*		1525.68			AGSD
Isoptera	Kalotermitidae	*Glyptotermes*	*nakajimai*		870.42			AGSD
Isoptera	Kalotermitidae	*Neotermes*	*koshunensis*		1828.86			AGSD
Isoptera	Mastotermitidae	*Mastotermes*	*darwiniensis*		1271.4			AGSD
Isoptera	Rhinotermitidae	*Coptotermes*	*formosanus*		909.54			AGSD
Isoptera	Rhinotermitidae	*Coptotermes*	*formosanus*		841.08			AGSD
Isoptera	Rhinotermitidae	*Reticulitermes*	*flavipes*		1046.46			AGSD
Isoptera	Rhinotermitidae	*Reticulitermes*	*speratus kyushuensis*		1046.46			AGSD
Isoptera	Rhinotermitidae	*Reticulitermes*	*speratus speratus*		987.78			AGSD
Isoptera	Rhinotermitidae	*Reticulitermes*	*yaeyamanus*		978			AGSD
Isoptera	Termitidae	*Nasutitermes*	*takasagoensis*		1643.04			AGSD
Isoptera	Termitidae	*Odontotermes*	*formosanus*		1447.44			AGSD
Isoptera	Termitidae	*Pericapritermes*	*nitobei*		1858.2			AGSD
Isoptera	Termopsidae	*Hodotermopsis*	*sjostedti*		1242.06			AGSD
Isoptera	Termopsidae	*Zootermopsis*	*nevadensis*		567.24			AGSD
Mantodea	Hymenopodidae	*Acromantis*	*japonica*		4430.34			AGSD
Mantodea	Mantidae	*Litaneutria*	*sp.*		3246.96			AGSD
Mantodea	Mantidae	*Stagmomantis*	*carolina*		3863.1			AGSD
Mantodea	Mantidae	*Statilia*	*maculata*		2982.9			AGSD
Mantodea	Mantidae	*Tenodera*	*aridifolia*		2855.76			AGSD
Orthoptera	Acrididae	*Acrida*	*conica*		10581.96			AGSD
Orthoptera	Acrididae	*Acrida*	*conica*		12273.9			AGSD
Orthoptera	Acrididae	*Ailopus*	*thalassinus*		6533.04			AGSD
Orthoptera	Acrididae	*Austroicetes*	*pusilla*		6151.62			AGSD
Orthoptera	Acrididae	*Caledia*	*captiva*		10660.2			AGSD
Orthoptera	Acrididae	*Campylacantha*	*olivacea*		6425.46			AGSD
Orthoptera	Acrididae	*Chorthippus*	*apicalis*		12332.58			AGSD
Orthoptera	Acrididae	*Chorthippus*	*binotatus binotatus*		10669.98			AGSD
Orthoptera	Acrididae	*Chorthippus*	*brunneus*		8361.9			AGSD
Orthoptera	Acrididae	*Chorthippus*	*brunneus*		9251.88			AGSD
Orthoptera	Acrididae	*Chorthippus*	*brunneus*		9926.7			AGSD
Orthoptera	Acrididae	*Chorthippus*	*cf. binotatus*		10122.3			AGSD
Orthoptera	Acrididae	*Chorthippus*	*dorsatus*		8156.52			AGSD
Orthoptera	Acrididae	*Chorthippus*	*jacobsi*		10601.52			AGSD
Orthoptera	Acrididae	*Chorthippus*	*jucundus*		11618.64			AGSD
Orthoptera	Acrididae	*Chorthippus*	*longicornis*		8391.24			AGSD
Orthoptera	Acrididae	*Chorthippus*	*nevadensis*		11276.34			AGSD
Orthoptera	Acrididae	*Chorthippus*	*parallelus*		12039.18			AGSD
Orthoptera	Acrididae	*Chorthippus*	*parallelus*		13066.08			AGSD
Orthoptera	Acrididae	*Chorthippus*	*parallelus*		13525.74			AGSD
Orthoptera	Acrididae	*Chorthippus*	*parallelus*		14396.16			AGSD
Orthoptera	Acrididae	*Chorthippus*	*scalaris*		14396.16			AGSD
Orthoptera	Acrididae	*Chorthippus*	*vagans*		8449.92			AGSD
Orthoptera	Acrididae	*Chorthippus*	*vagans*		8489.04			AGSD
Orthoptera	Acrididae	*Chortoicetes*	*terminifera*		5858.22			AGSD
Orthoptera	Acrididae	*Chortoicetes*	*terminifera*		7061.16			AGSD
Orthoptera	Acrididae	*Cryptobothrus*	*chrysophorus*		9163.86			AGSD
Orthoptera	Acrididae	*Eyprepocnemis*	*plorans*		9486.6			AGSD
Orthoptera	Acrididae	*Gastrimargus*	*musicus*		8811.78			AGSD
Orthoptera	Acrididae	*Gomphocerus*	*sibiricus*		8753.1			AGSD
Orthoptera	Acrididae	*Heteracris*	*adspersus*		6200.52			AGSD
Orthoptera	Acrididae	*Humbe*	*tenuicornis*		8029.38			AGSD
Orthoptera	Acrididae	*Locusta*	*migratoria*		5163.84			AGSD

Orthoptera	Acrididae	*Locusta*	*migratoria*		5349.66			AGSD
Orthoptera	Acrididae	*Locusta*	*migratoria*		5956.02			AGSD
Orthoptera	Acrididae	*Locusta*	*migratoria*		6132.06			AGSD
Orthoptera	Acrididae	*Locusta*	*migratoria*		6210.3			AGSD
Orthoptera	Acrididae	*Locusta*	*migratoria*		6298.32			AGSD
Orthoptera	Acrididae	*Macrotona*	*australis*		8303.22			AGSD
Orthoptera	Acrididae	*Melanoplus*	*differentialis*		3755.52			AGSD
Orthoptera	Acrididae	*Melanoplus*	*differentialis*		6092.94			AGSD
Orthoptera	Acrididae	*Melanoplus*	*differentialis*		6875.34			AGSD
Orthoptera	Acrididae	*Melanoplus*	*sanguinipes*		5701.74			AGSD
Orthoptera	Acrididae	*Myrmeleotettix*	*maculatus*		11872.92			AGSD
Orthoptera	Acrididae	*Myrmeleotettix*	*maculatus*		12381.48			AGSD
Orthoptera	Acrididae	*Myrmeleotettix*	*maculatus*		13085.64			AGSD
Orthoptera	Acrididae	*Omocestus*	*viridulus*		12870.48			AGSD
Orthoptera	Acrididae	*Peakesia*	*hospita*		10239.66			AGSD
Orthoptera	Acrididae	*Phaulacridium*	*vittatum*		10493.94			AGSD
Orthoptera	Acrididae	*Podisma*	*pedestris*		16557.54			AGSD
Orthoptera	Acrididae	*Schistocerca*	*cancellata*		9281.22			AGSD
Orthoptera	Acrididae	*Schistocerca*	*gregaria*		8361.9			AGSD
Orthoptera	Acrididae	*Schistocerca*	*gregaria*		8518.38			AGSD
Orthoptera	Acrididae	*Schistocerca*	*gregaria*		8762.88			AGSD
Orthoptera	Acrididae	*Schistocerca*	*paranensis*		8440.14			AGSD
Orthoptera	Acrididae	*Schizobothrus*	*flavovittatus*		7335			AGSD
Orthoptera	Acrididae	*Stauroderus*	*scalaris*		15980.52			AGSD
Orthoptera	Acrididae	*Valanga*	*irregularis*		9232.32			AGSD
Orthoptera	Eumasticidae	*Warramaba*	*virgo*		3667.5			AGSD
Orthoptera	Eumasticidae	*Warramaba*	*virgo*		3912			AGSD
Orthoptera	Gryllacrididae	*Ceuthophilus*	*stygius*		9339.9			AGSD
Orthoptera	Gryllidae	*Acheta*	*domesticus*		1956			AGSD
Orthoptera	Gryllidae	*Acheta*	*domesticus*		1956			AGSD
Orthoptera	Gryllidae	*Acheta*	*domesticus*		1956			AGSD
Orthoptera	Gryllidae	*Acheta*	*domesticus*		1956			AGSD
Orthoptera	Gryllidae	*Acheta*	*domesticus*		2327.64			AGSD
Orthoptera	Gryllidae	*Gryllus*	*pennsylvanicus*		1956			AGSD
Orthoptera	Gryllidae	*Gryllus*	*pennsylvanicus*		2014.68			AGSD
Orthoptera	Gryllidae	*Gryllus*	*pennsylvanicus*		2621.04			AGSD
Orthoptera	Gryllidae	*Hadenoecus*	*subterraneus*		1515.9			AGSD
Orthoptera	Gryllidae	*Laupala*	*cerasina*		1887.54			AGSD
Orthoptera	Gryllidae	*Oecanthus*	*niveus*		1672.38			AGSD
Orthoptera	Gryllotalpidae	*Neoscapteriscus*	*borellii*		3334.98			AGSD
Orthoptera	Tettigoniidae	*Conocephalus*	*sp.*		2777.52			AGSD
Orthoptera	Tettigoniidae	*Neoconocephalus*	*triops*		7442.58			AGSD
Orthoptera	Tridactylidae	*Unknown*	*sp.*		2572.14			AGSD
Phasmatodea	Pseudophasmatidae	*Anisomorpha*	*buprestoides*		2904.66			AGSD
Phasmatodea	Timematidae	*Timema*	*cristinae*		1330.08			AGSD
Phasmatodea	Heteronemiidae	*Diapheromera*	*femorata*		2493.9			AGSD
Phasmatodea	Phasmatidae	*Bacillus*	*atticus atticus*		2249.4			AGSD
Phasmatodea	Phasmatidae	*Bacillus*	*atticus caprai*		2180.94			AGSD
Phasmatodea	Phasmatidae	*Bacillus*	*atticus carius*		2875.32			AGSD
Phasmatodea	Phasmatidae	*Bacillus*	*atticus cyprius*		2396.1			AGSD
Phasmatodea	Phasmatidae	*Bacillus*	*grandii grandii*		2552.58			AGSD
Phasmatodea	Phasmatidae	*Bacillus*	*grandii grandii*		2112.48			AGSD
Phasmatodea	Phasmatidae	*Bacillus*	*rossius redtenbacheri*		2122.26			AGSD
Phasmatodea	Phasmatidae	*Bacillus*	*rossius redtenbacheri*		1907.1			AGSD
Phasmatodea	Phasmatidae	*Bacillus*	*whitei*		2220.06			AGSD
Phasmatodea	Phasmatidae	*Extatosoma*	*tiaratum*		7824			AGSD
Zoraptera	Zorotypidae	*Zorotypus*	*hubbardi*		1848.42			AGSD

Table S3 accession numbers of the sequences used for the inference of the phylogeny of Polyneoptera

Taxa name	COI	COX	ND4	X18S	X28S.A	X28S.B
Acanthops falcata	KU507627	KU528759	KU507927	KU320376	-	KU320480
Acanthoxyla geisovii	EU492959	EU492986	-	-	-	-
Acanthoxyla inermis	-	-	-	-	-	EU543518
Acrophylla titan	FJ474258	FJ474335	-	-	-	-
Amitermes darwini	-	EF442698	-	-	-	-
Amitermes germanus	-	EF442703	-	-	-	-
Amitermes parvus	-	EF442704	-	-	-	-
Anechura sp	-	-	-	KC413722	-	-
Anisolabis maritima	MF468289	AB005470	-	-	-	-
Antemna rapax	EF383875	EF384003	FJ802557	EF383553	-	EF383716
Antistia sp	-	-	-	FJ806471	-	-
Archimandrita tessellata	JN615372	DQ874263	-	DQ874110	DQ874197	-
Archimantis sobrina	EF383802	-	FJ802427	EF383602	-	AY491222
Arixenia esau	-	-	-	KX069014	-	-
Atrachelacris sp	AY014360	-	-	-	-	-
Bacillus atticus	-	AY185558	-	KF256446	-	KF256329
Bacillus grandii benazzii	-	AF148314	-	-	-	-
Bacillus grandii grandii	-	AF148301	-	-	-	-
Bacillus lynceorum	-	AF038214	-	-	-	-
Bacillus rossius	-	-	-	-	-	AY125320
Baculum sp	-	-	-	AY121172	-	-
Bifiditermes improbus	-	AF189079	-	-	-	-
Blaberus discoidalis	KF372514	AB014063	-	-	-	-
Blaberus giganteus	-	AB014064	-	-	-	-
Blatta orientalis	KP986400	DQ874267	FJ802410	AY521830	-	AY521741
Blattella bisignata	KT271843	-	-	-	-	-
Blattella germanica	AY176057	DQ874268	-	FJ806322	DQ874201	FJ806519
Blattella sauteri	KY349679	-	-	-	-	-
Bolbe pallida	FJ802759	-	FJ802436	FJ806343	-	FJ806541
Byrsotria fumigata	JN615375	DQ874269	-	DQ874117	DQ874202	-
Calineuria sp	-	-	-	AY521881	-	-
Callimantis antillarum	MF414717	-	-	MF414712	-	MF414715
Carausius morosus	FJ474268	FJ474344	-	AY121170	-	AY125310
Chaetospania sp	-	-	-	KX069010	-	-
Cheddikulama straminea	KT316265	KT316267	-	-	-	-
Chlorus bolivianus	FJ829333	-	-	-	-	-
Choeradodis rhombicollis	EF383805	-	FJ802431	EF383481	-	AY491226
Cliomantis cornuta	FJ802777	-	FJ802470	FJ806360	-	AY491264
Clitarchus hookeri	KF383507	EU492999	-	-	-	EU543521
Clonopsis felicitatis	-	GQ370542	-	-	-	-
Clonopsis gallica	-	AF096287	-	-	-	-
Clonopsis maroccana	-	GQ370575	-	-	-	-
Clonopsis soumiae	-	GQ370529	-	-	-	-
Coptotermes acinaciformis	HQ878425	FJ384631	-	DQ882634	DQ441893	-
Crenetermes albotarsalis	-	DQ442108	-	-	DQ441898	-
Creobroter laevicollis	FJ802780	FJ806890	FJ802488	FJ806363	-	FJ806562
Cryptocercus primarius	-	-	-	-	KU312232	-
Cryptocercus punctulatus	KY241441	AB005462	-	AY521829	DQ441902	AY521739
Cryptocercus relictus	-	KF855906	-	JX091733	KU312247	-
Cryptotermes austrinus	-	AF189081	-	-	-	-
Cryptotermes brevis	EU253840	EU253879	FJ802415	EU253783	-	FJ806528
Cryptotermes cynocephalus	-	AF189083	-	-	-	-
Cryptotermes domesticus	-	JQ678601	-	-	-	-
Cryptotermes dudleyi	-	FN377808	-	-	-	-
Cryptotermes primus	-	AF189090	-	-	-	-
Cryptotermes queenslandis	-	AF189092	-	-	-	-
Cryptotermes secundus	-	DQ442111	-	DQ882635	DQ441901	-
Ctenomorpha sp	-	FJ474350	-	-	-	-
Cubitermes sp	-	-	-	-	DQ441903	-

Deiphobe sp	-	-	-	-	-	KP340217
Deropeltis erythrocephala	-	DQ874271	-	DQ874121	-	-
Dichromatos lilloanus	FJ829336	-	-	-	-	-
Dichromorpha sp	-	-	-	KM853205	-	-
Dichroplus conspersus	DQ083454	-	-	-	-	-
Dichroplus democraticus	DQ083455	-	-	-	-	-
Dichroplus elongatus	KY595084	-	-	-	-	-
Dichroplus exilis	KY595085	-	-	-	-	-
Dichroplus fuscus	KY595086	-	-	-	-	-
Dichroplus maculipennis	KY595088	-	-	-	-	-
Dichroplus paraelongatus	KY595089	-	-	-	-	-
Dichroplus patruelis	DQ083458	-	-	-	-	-
Dichroplus pratensis	DQ083459	-	-	-	-	-
Dichroplus schulzi	DQ083460	-	-	-	-	-
Dichroplus silveiraguidoi	DQ083461	-	-	-	-	-
Dichroplus vittatus	KY595090	-	-	-	-	-
Didymocorypha lanceolata	EF383847	-	FJ802502	EF383524	-	EF383687
Diploptera punctata	JN615379	DQ874273	-	DQ874123	DQ874207	-
Diponthus sp	-	-	-	KM853222	-	-
Drepanotermes septentrionalis	-	EF442705	-	-	-	-
Dystacta alticeps	FJ802826	FJ806939	FJ802592	EF383571	-	EF383737
Ectobius pallidus	-	DQ874276	-	DQ874126	DQ874210	-
Embia nuragica	JQ907059	-	-	JQ907235	-	JQ906996
Embia ramburi	-	-	-	JQ907247	-	-
Embia tyrrhenica	JQ907057	-	-	JQ907233	-	JQ906994
Empusa sp	-	-	FJ802545	-	-	-
Ephelotermes melachoma	-	EF442709	-	-	-	-
Ephelotermes taylori	-	EF442710	-	-	-	-
Epilampra sp	-	-	-	KY497641	-	-
Eublaberus distanti	KF372518	KF372541	-	EU367508	-	-
Eublaberus posticus	JN615376	DQ874281	-	DQ874131	DQ874215	-
Euborellia moesta	-	AF140545	-	-	-	-
Eurycotis floridana	JN615395	DQ874283	-	DQ874133	-	-
Extatosoma tiaratum	KJ201987	KJ024574	-	AY121155	-	AY125295
Forficula auricularia	MF462143	-	-	Z97594	-	-
Galiblatta sp	-	-	-	KY497640	-	-
Galloisiana nipponensis	-	KC142671	-	-	-	-
Glyptotermes brevicornis	-	AF189096	-	-	-	-
Glyptotermes iridipennis	-	AF189097	-	-	-	-
Gongylus sp	-	-	FJ802421	-	-	-
Gonolabis sp	-	-	-	KC413693	-	-
Gromphadorhina portentosa	KF372519	DQ181523	-	Z97592	-	EF383626
Grylloblatta sp	-	KP972434	-	-	-	-
Haploembia solieri	JQ907039	-	-	JQ907206	-	-
Harpagomantis tricolor	KR360618	-	-	KR360562	-	KR360595
Hemimerus sp	-	-	-	JQ714388	-	-
Hemithyrsocera sp	KY349565	-	-	-	-	-
Hestiasula sp	-	-	FJ802734	-	-	-
Hierodula patellifera	JF700167	-	-	-	-	-
Humbertiella similis	EF383834	EF383961	FJ802484	EF383511	-	EF383672
Hyalopteryx rufipennis	-	-	-	KM853210	-	KM853480
Incisitermes sp	-	-	-	GQ337715	-	-
Iris sp	-	-	FJ802599	-	-	-
Isoperla grammatica	KU955920	-	-	-	-	-
Kalotermes flavicollis	EU253842	GU931799	-	EU253785	-	FJ806529
Karoophasma biedouwense	-	KF855912	-	-	-	-
Kongobatha sp	-	-	-	FJ806473	-	-
Labidura riparia	JN241998	AF140544	-	AY707356	-	-
Lamproblatta albipalpus	JN615394	KF855913	-	KF855836	-	-

Lanxoblatta emarginata	KF372522	KF372544	-	EU367509	-	-
Leiotettix flavipes	FJ829337	-	-	-	-	-
Leiotettix pulcher	DQ083464	-	-	-	-	-
Leiotettix viridis	AY014353	-	-	-	-	-
Leptynia attenuata	-	AF241441	-	-	-	-
Leptynia caprai	-	AF241431	-	-	-	-
Leptynia montana	-	AF241416	-	-	-	-
Leptysma sp	-	-	-	KM853199	-	-
Ligaria sp	-	-	FJ802551	-	-	-
Liturgusa cursor	KU507661	KU528782	KU507962	KU320411	-	KU320516
Liturgusa maya	KU507664	KU528783	FJ802555	KU320413	-	KU320518
Loboptera decipiens	KF372524	DQ874297	-	KF372502	-	-
Lobopterella dimidiatipes	KX053864	AB005905	-	-	-	-
Lophotermes septentrionalis	-	EF442708	-	-	-	-
Macrognathotermes sunteri	-	DQ442158	-	-	DQ441947	-
Macropanesthia rhinoceros	-	DQ874299	-	DQ874152	DQ874230	-
Macrotermes bellicosus	AY127702	JF923231	-	-	-	-
Mantis religiosa	FJ802846	KP639994	FJ802419	AY859586	-	EF383633
Marellia remipes	-	-	-	KM853256	-	KM853434
Margattea sp	KY349621	-	-	-	-	-
Mastotermes darwiniensis	JN615367	EU253885	-	DQ882638	-	EF383632
Microcerotermes boreus	-	EF442696	-	-	-	-
Microcerotermes nervosus	-	EF079031	-	-	-	-
Microtermes sp	-	-	-	-	JQ429105	-
Miomantis sp	-	-	FJ802627	-	-	-
Nala lividipes	-	-	-	AY707362	-	-
Nasutitermes graveolus	-	DQ442185	-	-	DQ441974	-
Nasutitermes longipennis	-	DQ442190	-	-	DQ441978	-
Nasutitermes triodiae	-	DQ442197	-	-	DQ441986	-
Nauphoeta cinerea	JN615381	DQ874301	-	KF372504	DQ874233	-
Neotermes insularis	-	AF189105	-	-	-	-
Nesogaster sp	-	-	-	AY707358	-	-
Odontotermes sp	-	-	-	EU253801	-	-
Oligonyx sp	-	-	KU508002	-	-	-
Oligotoma sp	-	-	-	JQ907216	-	-
Ommatolampis sp	-	-	-	KM853267	-	-
Ommexecha sp	-	-	-	KM853269	-	-
Orthodera sp	-	-	FJ802422	-	-	-
Orxines sp	-	-	-	AY121153	-	-
Panchlora nivea	JN615382	-	-	-	-	-
Panchlora viridis	-	DQ181534	-	-	-	-
Parathespis humbertiana	-	-	FJ802660	EF383616	-	EF383782
Paulinia acuminata	-	-	-	KM853257	-	KM853433
Pelmatosilpha sp	-	-	-	EU253773	-	-
Pericapritermes sp	-	-	-	-	DQ442004	-
Periplaneta americana	KR144773	M83971	-	AF370792	-	-
Periplaneta australasiae	JN615391	DQ874310	-	DQ874169	DQ874242	-
Periplaneta brunnea	MG572238	DQ874311	-	DQ874170	-	-
Periplaneta fuliginosa	MG458948	DQ874312	-	DQ874171	-	-
Periplaneta japonica	KC407710	-	-	KC413768	-	-
Perla abdominalis	MF458789	-	-	-	-	-
Perla marginata	KF492799	-	-	-	-	-
Perlodes sp	-	-	-	EF622774	-	-
Phalces sp	-	KT426619	-	-	-	-
Phobaeticus sp	-	-	-	AY121184	-	-
Phoetalia sp	-	-	-	KF372509	-	-
Phyllium bioculatum	-	KJ024570	-	Z97575	-	AY125301
Podacanthus wilkinsoni	GQ927386	GQ927422	-	-	-	-
Polyspilota aeruginosa	FJ802847	GU064726	FJ802575	EF383478	-	FJ806633
Porotermes adamsoni	-	LC193947	-	-	-	-
Prionolopha serrata	MF682223	-	-	-	-	-

Procryptotermes australiensis	-	AF189107	-	-	-	-
Procubitermes sp	-	-	-	-	DQ441918	-
Protermes sp	-	-	-	-	DQ442017	-
Pseudacanthops sp	-	-	KU508010	-	-	-
Pseudacanthotermes militaris	AY127731	DQ442233	-	DQ882644	DQ442020	-
Pseudomiopteryx infuscata	KU507710	-	KU508012	KU320463	-	KU320566
Pseudophasma sp	-	KJ024573	-	-	-	-
Pseudoscopas nigrigena	FJ829342	-	-	-	-	-
Pycnoscelus surinamensis	JN615383	DQ874325	-	DQ874185	DQ874252	-
Reticulitermes flavipes	AY027469	EU689011	-	-	-	-
Reticulitermes lucifugus	KM245729	JQ231192	-	-	-	-
Reticulitermes urbis	KM245722	JQ231191	-	-	-	-
Rhabdoblatta sp	-	AB007543	-	-	-	-
Ronderosia sp	DQ083468	-	-	-	-	-
Schedorhinotermes lamanianus	-	DQ442239	-	DQ882645	DQ442025	-
Schistocerca cancellata	KY980925	KY980962	KY981176	-	-	-
Schistocerca flavofasciata	KY980916	KY980953	-	-	-	-
Schistocerca pallens	KY980891	KY980928	KY981153	KM853186	-	KM853504
Schizocephala bicornis	EF383831	EF383958	FJ802480	EF383508	-	EF383669
Scotussa daguerrei	DQ083469	-	-	-	-	-
Scotussa impudica	KY595091	-	-	-	-	-
Scotussa lemniscata	FJ829338	-	-	-	-	-
Sipyloidea sipylus	FJ474324	FJ474393	-	AY121181	-	AY125321
Sphodromantis sp	-	-	FJ802430	-	-	-
Sphodropoda sp	-	-	FJ802670	-	-	-
Statilia maculata	FJ802905	AB006435	FJ802477	FJ806503	-	FJ806717
Stolotermes sp	-	-	-	EU253798	-	-
Supella longipalpa	FJ802748	FJ806878	FJ802414	EF383467	-	FJ806526
Symploce sp	-	-	-	DQ874188	-	-
Symplocodes sp	KY349568	-	-	-	-	-
Tenodera aridifolia	-	GU064747	-	AF423805	-	AY125282
Tenodera australasiae	-	GU064735	-	-	-	-
Tenodera superstitiosa	-	GU064753	-	-	-	-
Thesprotia graminis	EF383826	EF383952	FJ802473	EF383502	-	EF383662
Thoracotermes macrothorax	-	DQ442254	-	-	DQ442040	-
Timema poppensis	HQ184611	-	-	-	-	-
Timema shepardi	HQ184658	-	-	-	-	-
Trimerotropis ochraceipennis	JQ513038	-	-	-	-	-
Trimerotropis pallidipennis	JQ513036	GU476998	-	-	-	-
Tropidostethus sp	-	-	-	KM853196	-	-
Tuberculitermes bycanistes	-	DQ442258	-	-	DQ442044	-
Tumulitermes pastinator	-	DQ442261	-	-	DQ442047	-
Unguitermes sp	-	-	-	-	DQ442050	-
Vates sp	-	-	FJ802429	-	-	-
Xestoblatta sp	-	-	-	KF372512	-	-
Xyleus insignis	MF682248	-	-	-	-	-
Xyleus laevipes	MF682234	-	-	-	-	-
Xyleus modestus	KJ889693	-	-	KM853221	-	KM853469
Zoniopoda hempeli	MF682228	-	-	-	-	-
Zoniopoda omnicolor	MF682227	-	-	-	-	-
Zoniopoda tarsata	MF682225	-	-	-	-	-
Zootermopsis angusticollis	MF477188	DQ442267	-	AY859615	-	-
Zootermopsis nevadensis	JN615368	EU253894	AB936819	EU253799	-	-

Table S4 The mean age and standard deviations applied to specified nodes in our beast analysis.

Node	Mean	Standard Deviation
Blattodea (Including Isoptera)	197	28
Isoptera	136	4
Phasmatodea + Embiidina	164	31
Plecoptera	269	40
Dermaptera	302	46
Orthoptera	248	36
Notoptera	204	34

Table S5 95% Highest Posterior Density distribution for chromosome fissions, fusions, and polyploidy of the six analyses.

Order	Fission	Fusion	Polyploidy
Blattodea (including Isoptera)	0.063 - 0.257	0.040 - 0.243	0.000 - 0.005
Blattodea (excluding Isoptera)	0.173 - 0.604	0.202 - 0.619	0.000 - 0.010
Isoptera	0.002 - 0.100	0.019 - 0.114	0.000 - 0.006
Mantodea	0.000 - 0.329	0.000 - 0.737	0.000 - 0.690
Orthoptera	0.000 - 0.008	0.010 - 0.041	0.034 - 0.175
Phasmatodea	0.000 - 0.342	0.275 - 0.639	0.006 - 0.074

Table S6 Transition rates and mean number of transitions obtained from the stochastic mapping of sex chromosome evolution model

Transition	Mean Rate (95% credible interval	Mean number of transitions
XO to XY	0.0020 (0.0015 - 0.0026)	15.3
XY to XO	0.0021 (0.0010 - 0.0036)	6.7

Table S7 Transition rates and the mean number of transitions from sexual to asexual reproduction from stochastic mapping. The transition rate of parthenogenesis to sexual reproducing was set to zero.

Mean Rate (95% credible interval	Mean number of transitions
0.0063 (0.0052 - 0.0078)	9.3

References

1. Blackmon H, Ross L, Bachtrog D. Sex determination, sex chromosomes, and karyotype evolution in insects. Journal of Heredity. 2017;108(1):78-93.
2. Consortium ToS. Tree of Sex: A database of sexual systems. Scientific Data. 2014;1.
3. Smith SA, Walker JF. Py phlawd: A python tool for phylogenetic dataset construction. Methods in Ecology and Evolution. 2019;10(1):104-8.
4. Blackmon H, Adams R. EvobiR: tools for comparative analyses and teaching evolutionary biology. Crossref; 2015.
5. Katoh K, Rozewicki J, Yamada KD. MAFFT online service: multiple sequence alignment, interactive sequence choice and visualization. Briefings in bioinformatics. 2019;20(4):1160-6.
6. Castresana J. GBLOCKS: selection of conserved blocks from multiple alignments for their use in phylogenetic analysis. Version 091 b Copyrighted by J Castresana, EMBL. 2002.
7. Aberer AJ, Krompass D, Stamatakis A. Pruning rogue taxa improves phylogenetic accuracy: an efficient algorithm and webservice. Systematic biology. 2013;62(1):162-6.
8. Rabosky DL. No substitute for real data: a cautionary note on the use of phylogenies from birth–death polytomy resolvers for downstream comparative analyses. Evolution. 2015;69(12):3207-16.
9. Stamatakis A. RAxML version 8: a tool for phylogenetic analysis and post-analysis of large phylogenies. Bioinformatics. 2014;30(9):1312-3.
10. Miller MA, Pfeiffer W, Schwartz T, editors. Creating the CIPRES Science Gateway for inference of large phylogenetic trees. 2010 gateway computing environments workshop (GCE); 2010: Ieee.
11. Maddison W, Maddison D. Mesquite: a modular system for evolutionary analysis. Version 3.51. . 2018.
12. Bouckaert R, Heled J, Kühnert D, Vaughan T, Wu C-H, Xie D, et al. BEAST 2: a software platform for Bayesian evolutionary analysis. PLoS Comput Biol. 2014;10(4):e1003537.
13. Misof B, Liu S, Meusemann K, Peters RS, Donath A, Mayer C, et al. Phylogenomics resolves the timing and pattern of insect evolution. Science. 2014;346(6210):763-7.
14. Rambaut A, Drummond AJ, Xie D, Baele G, Suchard MA. Posterior summarization in Bayesian phylogenetics using Tracer 1.7. Systematic biology. 2018;67(5):901.
15. Sanderson MJ, Boss D, Chen D, Cranston KA, Wehe A. The PhyLoTA Browser: processing GenBank for molecular phylogenetics research. Systematic Biology. 2008;57(3):335-46.
16. Johnston JS, Bernardini A, Hjelmen CE. Genome size estimation and quantitative cytogenetics in insects. Insect Genomics: Springer; 2019. p. 15-26.
17. Hanrahan SJ, Johnston JS. New genome size estimates of 134 species of arthropods. Chromosome Research. 2011;19(6):809.
18. Glick L, Mayrose I. ChromEvol: assessing the pattern of chromosome number evolution and the inference of polyploidy along a phylogeny. Molecular Biology and Evolution. 2014;31(7):1914-22.
19. Mayrose I, Barker MS, Otto SP. Probabilistic models of chromosome number evolution and the inference of polyploidy. Systematic biology. 2010;59(2):132-44.

20. Zenil-Ferguson R, Burleigh JG, Ponciano JM. chromploid: An R package for chromosome number evolution across the plant tree of life. Applications in plant sciences. 2018;6(3).
21. Freyman WA, Höhna S. Cladogenetic and anagenetic models of chromosome number evolution: a Bayesian model averaging approach. Systematic Biology. 2018;67(2):195-215.
22. Blackmon H, Justison J, Mayrose I, Goldberg EE. Meiotic drive shapes rates of karyotype evolution in mammals. Evolution. 2019;73(3):511-23.
23. White M. Animal Cytology and Evolution. London, UK: Cambridge University Press; 1973
24. Bush GL, Case S, Wilson A, Patton J. Rapid speciation and chromosomal evolution in mammals. Proceedings of the National Academy of Sciences. 1977;74(9):3942-6.
25. Coyne JA. Correlation between heterozygosity and rate of chromosome evolution in animals. The American Naturalist. 1984;123(5):725-9.
26. Ross L, Blackmon H, Lorite P, Gokhman VE, Hardy NB. Recombination, chromosome number and eusociality in the Hymenoptera. Journal of evolutionary biology. 2015;28(1):105-16.
